# Potential of Benchtop NMR for the Determination of Polymer Molar Masses, Molar Mass Distributions, and Chemical Composition Profiles by Means of Diffusion‐Ordered Spectroscopy, DOSY

**DOI:** 10.1002/marc.202400512

**Published:** 2024-08-06

**Authors:** Johanna Tratz, Marianne Gaborieau, Markus Matz, Michael Pollard, Manfred Wilhelm

**Affiliations:** ^1^ Institute of Chemical Technology and Polymer Chemistry Karlsruhe Institute of Technology (KIT) Engesserstraße 18 76131 Karlsruhe Germany

**Keywords:** bimodal polymer samples, block copolymers, chemical composition profiles, diffusion coefficient distributions, DOSY, low‐field NMR, polymer molar mass distribution

## Abstract

The determination of molar masses and their distributions is crucial in polymer synthesis and design. This work presents the current performance and limitations of diffusion‐ordered spectroscopy (DOSY) on a low‐field (benchtop) NMR spectrometer (at 90 MHz) as an alternative to size exclusion chromatography (SEC) for determining diffusion coefficient distributions (DCDs) and molar mass distributions (MMDs). After optimization for narrowly distributed homopolymers, MMDs obtained with inverse Laplace transformation (ILT) and log‐normal distribution are compared with average molar masses obtained with mono‐ and bi‐exponential fits, as well as MMDs obtained from SEC. This approach enables ILT to determine DCDs and MMDs even for bimodal homopolymers with fully spectrally overlapping signals and block copolymers with various chemical compositions, for which chemical composition profiles are determined. The feasibility of low‐field diffusion NMR with samples dissolved in non‐deuterated solvents is further demonstrated and methods for solvent suppression are discussed.

## Graphical abstract

This work presents the performance and limitations of diffusion‐ordered spectroscopy on a 90 MHz NMR spectrometer. It includes method optimization for fast polymer molar mass determination, evaluation of mathematical fitting models, and validation of various polymer samples including bimodal samples and block copolymers to obtain diffusion coefficient distributions, molar mass distributions, and chemical composition profiles.

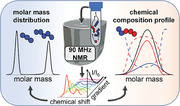

## 1 Introduction

The molar mass of polymers is directly related to their functional properties, like solid mechanical strength, melting temperature, and melt viscosity.^[^ ^1^ ^]^ Therefore, determining molar masses is of major interest for synthesizing and designing polymers. The molar mass distribution (MMD) is defined here as the mass distribution *W*(*M*), i.e., the weight fraction as the fundamental NMR signal is proportional to the number of monomer units. In addition to the complete functional form of *W*(*M*), two mean values are of particular importance for the correlation with macroscopic properties, namely the number‐averaged molar mass (*M*_n_) and the weight‐averaged molar mass (*M*_w_) which can be quantified from a weight MMD with:^[^ ^2^ ^]^

(1) $$\;M_{n}=\frac{\sum_{i}W\left(M_{i}\right)}{\sum_{i}\frac{W\left(M_{i}\right)}{M_{i}}}$$

(2) $$\;M_{w}=\frac{\sum_{i}W\left(M_{i}\right)\;M_{i\;}}{\sum_{i}W\left(M_{i}\right)}$$

where *M*_i_ is the molar mass and *W*(*M*_i_) is its corresponding probability at each point of the distribution. The dispersity, *Đ*, is used to express the distribution width:

(3) $$D=\frac{M_{w}}{M_{n}}$$

There are many techniques to measure different average molar masses, including light‐scattering,^[^ ^3^ ^]^ osmometry,^[^ ^4^ ^]^ and viscometry.^[^ ^5^ ^]^ The most common method is size exclusion chromatography (SEC, also called gel permeation chromatography, GPC).^[^ ^2^, ^6^, ^7^ ^]^ SEC is commonly used due to the availability of various detector systems, its wide accessibility, and the feasibility of obtaining MMDs. However, this method has some disadvantages, such as high solvent consumption (typically 10–60 mL per measurement), the need for specific columns depending on the sample and solvent, time‐consuming measurements, as well as the need for frequent calibration of the system.

A possible alternative for determining molar masses is by measuring diffusion coefficients with pulsed field gradient (PFG) NMR spectroscopy. A two‐dimensional presentation of the PFG NMR experiment is the spectroscopically‐resolved diffusion‐ordered spectroscopy (DOSY).^[^ ^8^ ^]^ Analytes in mixtures are discriminated by DOSY based on their chemical shift (1^st^ dimension), which is plotted against the self‐diffusion coefficients, *D* (2^nd^ dimension). The *M*_w_ of a molecule can be obtained from diffusion NMR measurements, as the NMR signal decay depends on the size (hydrodynamic radius) of a molecule and every monomer unit contributes equally. For details see Section 2. To calculate diffusion coefficient distributions (DCDs) and the corresponding MMDs of polymers, either univariate or multivariate approaches are necessary.^[^ ^9^ ^]^ Numerous determinations of average molar masses or MMDs on high‐field NMR spectrometers have been reported.^[^ ^10^, ^11^, ^12^, ^13^, ^14^, ^15^, ^16^, ^17^ ^]^ PFG NMR is a non‐invasive method, without special sample preparation and with low solvent consumption (<1 mL). Furthermore, the simultaneous study of NMR chemical shifts and *J*‐couplings is possible which provides more information about the sample and allows assigning and differentiating molecules with the same molar mass but different chemical structures. Although PFG NMR has advantages over SEC, the complexity and space requirements of high‐field NMR devices, along with ongoing operating costs and the availability of liquid helium and nitrogen, are significant drawbacks to its use. Therefore, small facilities or non‐NMR‐specialised research groups often cannot perform these NMR experiments. Alternatively, newly developed low‐field NMR spectrometers with permanent magnets up to 2.3 T (100 MHz, ^1^H) and a perpendicular sample orientation of the horizontal *B*_0_ field offer several advantages. The main benefits are their compact benchtop design (< 100 kg and < (0.5 m)^3^), with a *B*_0_ stray field <2 Gauss within the spectrometer, low operating costs as no cryogenic liquids (N_2_, He) are needed for cooling, and an easy setup.^[^ ^18^ ^]^ Furthermore, due to an external ^19^F‐lock even protonated solvents can be used without the addition of deuterated solvents. Various solvent suppression techniques enable the analysis of molecules in protonated solvents.^[^ ^19^ ^]^ In contrast, the current disadvantages of benchtop NMR spectrometers are lower sensitivity, and resolution, as well as weaker gradient strengths compared to high‐field instruments. Very recently average molar masses were also determined for polymers with PFG NMR at low field (80 MHz) with reduced gradient strengths and limited parameter optimization compared to the present study.^[^ ^20^ ^]^

This study aims to examine the potential and limitations of benchtop spectrometers in the context of diffusion NMR to determine MMDs and assess their dispersity, as an alternative to SEC or diffusion NMR at high field. This article consists of four parts: 1) Optimization of parameters for a fast and accurate molar mass determination up to 1000 kg·mol^−1^; 2) Comparison of different fitting models of the magnetization decay, including a monoexponential fit, a biexponential fit, a log‐normal distribution, and an inverse Laplace transformation (ILT); 3) Representation of DCDs and MMDs for complex samples, including bimodal samples and copolymers with their chemical composition profiles; and 4) Investigation of the feasibility of PFG measurements in protonated solvents. Polystyrene (PS) was selected for this study, in combination with poly(methyl methacrylate) (PMMA) in block copolymers.

## 2 Theoretical Background

Diffusion NMR is used to measure the self‐diffusion coefficient, *D*, caused by the spontaneous motion of molecules in liquids or gases (Brownian motion). According to the Stokes–Einstein Equation (Equation 4), the diffusion of spherical, independently moving particles is given by:

(4) $$D\;=\frac{kT}{6\pi \eta R_{H}}\;$$

where *k* is the Boltzmann constant, *T* is the absolute temperature, *η* is the solvent viscosity, and *R*_H_ is the particle's hydrodynamic radius.

**Figure 1:**
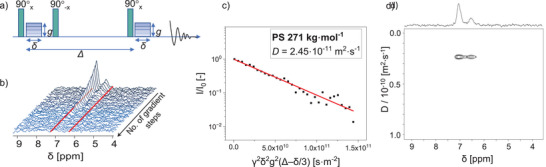
a) Schematic presentation of the used pulsed field gradient stimulated echo pulse sequence with *g*: gradient strength, *δ*: gradient pulse length, *∆*: diffusion time. b) Recorded ^1^H‐NMR spectra (within ≈11 min) of anionically synthesized PS (271 kg⋅mol^−1^), showing a decaying signal of the aromatic protons and integration range (6.3–7.2 ppm) of the peaks, indicated by red lines. c) Monoexponential fit for calculating the diffusion coefficient *D*. d) DOSY plot of the aromatic protons obtained with inverse Laplace transformation.

In ^1^H‐NMR spectroscopy, the diffusion coefficient can be measured through a series of one‐dimensional experiments with various gradient strengths (**Figure** **1**). Typical gradients are in the range of 0.1–10 T·m^−1^. The basic pulse sequence is a PFG‐Spin‐Echo experiment, although numerous variations and optimized pulse sequences have been developed, e.g., PFG‐stimulated echo (PFGSTE), bipolar pulse pair STE, bipolar pulse pair longitudinal eddy current delay, and the Oneshot pulse sequence.^[^ ^10^, ^21^ ^]^ During a gradient pulse, the molecules in the sample experience position‐dependent magnetic fields along an axis, resulting in different Larmor frequencies and leading to phase shifts after the application. Due to self‐diffusion during a delay time, only the phase of spins that have not changed position along this axis is refocused after a second read‐out gradient pulse. As a result, the intensities of the detected signals are attenuated with increasing gradient strengths and delay time due to a greater degree of dephasing across the sample.^[^ ^22^ ^]^

Stejskal and Tanner developed a monoexponential model to describe the signal decay and to extract the mean diffusion coefficient *D* as a function of the NMR parameter *q* ^[^ ^23^ ^]^:

(5) $$\frac{I}{I_{0}}=\exp\left(-qD\right)\;\mathrm{with}\;q=g^{2}\delta^{2}\gamma^{2}\left(\Delta -\frac{\delta }{3}\right)$$

where *I/I*_0_ is the ratio of the detected intensity to the signal intensity in the absence of the gradient, *g* is the gradient strength, *δ* is the duration of the gradient pulse, *∆* is the diffusion time and *γ* is the magnetogyric ratio of ^1^H (42.6 MHz·T^−1^). Various approaches were already exploited to determine *D*. The monoexponential decay function from Equation 5 is restricted to samples of uniform size and only allows the determination of the mean *D*. For non‐uniformly sized samples, like many polymers, several distinct *D*s can be calculated through either univariate or multivariate approaches, such as a biexponential fitting model,^[^ ^24^ ^]^ the log‐normal distribution,^[^ ^15^, ^25^ ^]^ or the inverse Laplace transformation,^[^ ^16^, ^26^, ^27^ ^]^ see Section 3.2.2 for detailed discussion.

## 3 Results and Discussion

### 3.1 Parameter Optimization for Diffusion Coefficient Determination

**Figure 2:**
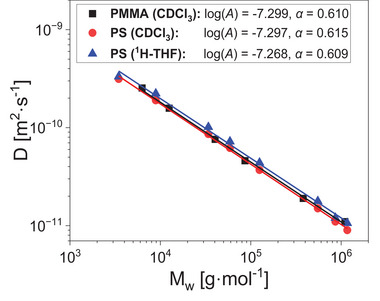
Calibration curves of uniform PS standards in CDCl_3_ (Section 3.2.1) or protonated THF (Section 3.4) and PMMA standards in CDCl_3_ (Section 3.3.2). See Table S1 (Supporting Information) for sample details. Fitted calibration parameters *A* and *α* are shown according to Equation 6 with *M*_w_ in g·mol^−1^ and *D* in m^2^·s^−1^. The squared correlation coefficient (R^2^) for all curves is greater than 0.99.

The precise and accurate determination of molar masses using PFG first requires a careful optimization of the diffusion parameters. These include **1)** the diffusion delay *∆*, **2)** the gradient pulse length *δ*, **3)** the number of gradient steps, and **4)** the sample concentration. Additionally, this method was optimized for a time‐efficient measurement by optimizing acquisition parameters such as **5)** the repetition time and **6)** the number of scans. For improvement in the signal‐to‐noise ratio (SNR) without increasing measurement time different apodization filter functions **7)** were investigated for the NMR spectra. PS550k, which was used for calibration (Table S1, Supporting Information), was selected for optimization as it is in the middle of the linear molar mass range of the NMR and SEC calibration curves (1–1000 kg·mol^−1^, see **Figure** **2**; Figure S1, Supporting Information).

**1)** During the diffusion delay *∆*, molecules move from their original position. The larger the molecule, the lower the diffusion coefficient *D* and the longer *∆* needed for an adequate signal decay. To ensure primarily diffusion processes influence this signal decay, *∆* is constrained by *T*_1_ of the fastest relaxing proton in the sample.^[^ ^22^ ^]^ **2)** Increasing the gradient pulse length *δ* leads to a greater dephasing of the spins. To calibrate over a wide range of molar masses, selecting appropriate *∆* and *δ* values for both larger and smaller molecules is important. Both parameters were varied independently and signal intensities were plotted against the gradient strength. Considering the strength of the signal decay and the correlation coefficient R^2^ as quality criteria, as well as the short *T*_1_ of macromolecules (from *T*_1 _ =  0.32 s for PS1200k to *T*_1 _ =  0.39 s for PS3k calibration standards, see Figure S2, Supporting Information), an upper limit of *∆*  =  300 ms was chosen for further measurements (Figure S3, Supporting Information). For *δ*, 5 ms was considered optimal, as the strongest signal decay and the best linear correlation between the signal decay and the gradient strength were observed (Figure S4, Supporting Information). Furthermore, enabling the gradient for an extended duration *δ* may harm the hardware,^[^ ^22^ ^]^ thus, this parameter should be varied with caution.

**3)** Experiments were then carried out with 16, 32, and 64 gradient steps with 10 replicates each. The relative standard deviation (RSD) of the measured *D*s for PS550k decreased from 3.1% for 16 steps to 1.4 %, and 1.3 % for 32 and 64 steps, respectively (Table S2, Supporting Information), thus subsequent experiments were carried out with 32 steps. The optimized diffusion parameters (*∆* and *δ*) were designed for a wide molecular weight range of 1–1000 kg⋅mol^−1^. However, the signal attenuation for molecules in the lower molar mass range (< 20 kg⋅mol^−1^) is very strong, resulting in a small number of data points at low gradient strengths. To ensure a reliable determination of *D*, 128 gradient steps were used for samples with molar masses lower than 20 kg⋅mol^−1^, resulting in more data points in the front part of the signal decay.

**4)** While conventional ^1^H NMR experiments favor high sample concentrations for sufficient sensitivity, PFG measurements for polymers should be carried out with dilute samples to reduce the impact of polymer concentration on viscosity and to avoid polymer‐polymer interactions prevalent above the critical overlap concentration, c*.^[^ ^12^, ^30^ ^]^ Due to lower magnetic field strengths and therefore lower sensitivity in benchtop NMR instruments, it is particularly important to determine the maximum possible sample concentration without a significant reduction of *D*. As c* is dependent on the molar mass (c*∼*M* ^‐0.8^, for linear coils in a good solvent^[^ ^31^ ^]^) and thus on *D*, diffusion measurements for different calibration standards (PS550k and PS1200k) at different concentrations (1–10 g·L^−1^) were conducted. The measured *Ds* fluctuated by up to 10 % from 1 to 2.5 g·L^−1^. This variation could be attributed to the low sample concentration and resulting low sensitivity of the NMR signals. Higher concentrations (up to 10 g·L^−1^) led to a decrease of *D*, due to mutual hindrance of the polymer chains and to even greater deviations from the *D* values at low concentrations (Figure S5, Supporting Information). Therefore, a concentration of 2.5 g·L^−1^ was chosen for further experiments to maximize sensitivity while maintaining a maximal deviation of 10 % of *D* at low concentrations. For molar mass determinations, *D* deviations due to concentration fluctuations will be eliminated, as the calibration and all subsequent measurements are conducted at the same concentration.

To optimize the sensitivity and measurement time of the NMR method **5**) the repetition time, **6)** the number of scans, and **7)** the apodization filter were adjusted. Since only the gradient strength varies between steps in the experiment, the signal loss through *T*_1_ and *T*_2_ is the same in all steps.^[^ ^22^ ^]^ Thus, the relaxation processes are independent of the diffusion conditions.

**5)** To minimize measurement time, the shortest possible repetition time was chosen that allowed for the technical execution of the pulse sequence: 2.5 s, which corresponds to about 6 *T*_1_ of the aromatic protons of PS3k.

**6)** The number of scans is also crucial for the experiment time, as SNR increases with the square root of the number of scans. Four replicate experiments with each 2, 4, 8, 16, or 32 scans per spectrum were performed (Table S3, Supporting Information). The relative precision of the *D*s, quantified by the residual standard deviation RSD, increased by a factor of nearly 4 between 2 and 8 scans (RSDs of 6.3 and 1.7 %, respectively), and only minimally further increased for 16 and 32 scans (RSD of 1.5 and 1.3 %, respectively). This suggests that white spectral noise is not the sole cause of uncertainty in diffusion measurements. Longer measurement times might lead to further uncertainties that scale linearly in time due to temperature fluctuations, variability of radio frequency and gradient pulses as well as changes in the decaying profile due to convection.^[^ ^32^ ^]^ Consequently, for further experiments 8 scans were chosen, leading with 32 steps to a total measurement time of 11 min 19 s.

**7)** To improve the SNR without increasing experiment time different apodization filters can be utilized. These filters are mathematical functions used to weight certain parts of the free induction decay, increasing SNR but often resulting in peak broadening. In this work, an exponential and a Gaussian filter were compared at different exponential decaying constants (*σ*) or standard deviations of the Gaussian curve respectively. The squared correlation coefficient R^2^ of the Stejskal‐Tanner plot and the increase in SNR in the NMR spectra were used to select the optimal apodization filter. These criteria are limited by the increase in peak width, which can cause the aromatic protons signal to overlap with that of the chloroform (solvent) at 7.26 ppm, resulting in the determination of inaccurate diffusion coefficients. Figure S6 (Supporting Information) shows the effect of different filter functions on the aromatic protons signal at 7.04 ppm in the spectrum at the first gradient step of the PFGSTE experiment, with the resulting SNR and the full width at half maximum (FWHM). The SNR is highest when using the strongest smoothing factor (*σ* = 5.0 Hz) for both the Gaussian filter (SNR = 51) and the exponential filter (SNR = 62). However, increasing *σ* also increases the FWHM, resulting in greater peak broadening with the exponential filter. Although *σ* = 5.0 Hz maximizes SNR, a value of *σ* = 2.0 Hz exponential filter was selected for further measurements, giving the highest SNR increase while not exceeding a 25 % increase in peak width. This ensured that the aromatic protons signal was sufficiently separated from the chloroform peak for integration. With increasing *σ*, R^2^ of the Stejskal‐Tanner plots increases from 0.82 (without filter) to 0.98 (Gauss 5.0 Hz) or 0.99 (exponential 5.0 Hz), resulting in a better linear correlation of the normalized intensity against the gradient strength with stronger smoothing factors (Figure S7, Supporting Information). Overall, an apodization of the NMR data with exponential 2.0 Hz led to a nearly four‐fold increase in SNR and an improvement in R^2^ from 0.82 to 0.98 compared to data without apodization.

### 3.2 Determination of Average Molar Masses and Molar Mass Distributions

#### 3.2.1 Molar Mass Calibration

While *M*_n_ can be determined using ^1^H‐NMR by end‐group analysis, *M*_w_ is directly accessible through the decaying ^1^H signals in a PFG experiment, since each monomer contributes equally to the signal. Thus, the measurement of the diffusion coefficient *D* is possible. In the case of uniform samples, *M*_w_ = *M*_n_. A power law relationship of *D* to *M*_w_ enables the calculation of weight average molar masses^[^ ^33^ ^]^:

(6) $$D=A\cdot {M_{w}}^{-\alpha }$$

where *A* and *α* are parameters that depend on the chemical nature of the polymer and solvent, the temperature, and branching if present. Therefore, to conduct molar mass determinations a calibration must be established for each molecule and solvent involved at the relevant temperature.

Calibrations were conducted with linear PS and PMMA standards over the range of 1 to 1000 kg·mol^‐1^ (Table S1, Supporting Information) in CDCl_3_ or protonated THF at 26.5 °C. Values of *D* determined with the optimized parameters described in the previous section exhibited a linear dependence on *M*_w_ on logarithmic scales, allowing the determination of *A* and *α* through Equation 6 in each case (Figure 2).

Both *A* and *α* varied only slightly with different polymer samples and solvents. The scaling exponent *α* provides information about the interactions between the polymer and the solvent. Values of ≈0.6 agree with literature values for PS and PMMA in CDCl_3_ and THF determined with PFG NMR (*α* = 0.52 to 0.62).^[^ ^15^, ^16^, ^34^ ^]^ Similar to the Flory exponent, this value shows that the polymers become fully solvated and interactions between the solvent and the polymers are maximized, which is characteristic of CDCl_3_ and THF as they both are good solvents for PS and PMMA.^[^ ^34^, ^35^ ^]^ When plotted in a double logarithmic graph, both polymers exhibit a linear dependency of *D* and *M*_w_ with R^2^ higher than 0.99, suggesting parameter settings that enable the determination of *M*_w_ for unknown samples by measuring *D* via PFG NMR.

#### 3.2.2 Comparison of Different Approaches for Diffusion Coefficient and Molar Mass Determination

A polymer sample is generally a mixture of many macromolecules with different molar masses. To characterize a polymer, it is therefore imperative to know its molar mass distribution (MMD), usually described through its statistical moments *M*_n_ and *M*_w_, as well as its dispersity *Đ*. The parameter *Đ* is related to the standard deviation of the distribution *σ*, as *σ*∼(*Đ*‐1)^1/2^; it is used to quantify the distribution's width.^[^ ^31^ ^]^ For uniform, monomodal samples a single diffusion coefficient, *D*, can be extracted from the exponential decay of the signal intensities (Equation 5) and *M*_w_ can be calculated using the scaling law (Equation 6); however, for non‐uniform samples the MMD is not directly accessible using this method. A two‐component polymer solution can be modeled with a biexponential fit, each polymer having a certain diffusion coefficient and thus a certain molar mass.^[^ ^24^, ^36^ ^]^ Equation 7 allows for the determination of the different diffusion coefficients (*D*_1_ and *D*_2_) of two fully spectrally overlapping components with relative weight fractions *X*_wt_ and 1‐*X*_wt_.

(7) $$\frac{I}{I_{0}}=X_{\mathrm{wt}}\;\exp\;\left(-qD_{1}\right)+\left(1-X_{\mathrm{wt}}\right)\;\exp\left(-qD_{2}\right)$$

In principle it would be possible to obtain a distribution from the knowledge of *D*_1_, *D*_2,_ and *X*_wt_, however, most polymer samples are composed of more than two components, which cannot be represented through a biexponential distribution. Determining such distributions is challenging because of the increased number of fitting parameters compared to uniform samples. Intensities can be modeled as a distribution of exponential functions. With Equation 5 the distribution of the diffusion coefficients P(*D*) follows:

(8) $$\frac{I}{I_{0}}=\underset{0}{\int^{\infty }}\exp\;\left(-\mathrm{qD}\right)P\left(D\right)\;dD$$

Methods to obtain these distributions are either through closed forms, such as the log‐normal distribution, which does not rely on numerical calculations, or by using functions that do not have an analytical form, like the inverse Laplace transformation (ILT).^[^ ^15^ ^]^ Equation 8 has the form of a Laplace transformation. To obtain information about the MMD, the diffusion coefficient distribution (DCD) must first be determined by doing an ILT and then converted into the MMD. For this Equation 6 was used to convert the *x*‐axis to molar masses and Equation 9 to convert the *y*‐axis into the mass distribution *W*(*M*) with the calibration parameters for PS in CDCl_3_ shown in Figure 2 ^[^ ^27^ ^]^:

(9) $$W\left(M\right)=P\left(D\right)\;\left|\frac{dD}{dM}\right|=\alpha \;A\;{M_{w}}^{-\alpha -1}\;P\left(D\right)$$

Instead of using the ILT approach a specific functional form for *P*(*D*) can also be used. Assuming *P*(*D*) has the shape of a log‐normal distribution *P*_LN_(*D*), the DCD can be obtained with:

(10) $$P_{\mathrm{LN}}\left(D\right)=\frac{1}{\sqrt{2\pi }\sigma_{D}D}\exp\left[\frac{-{\left(\log D-\log\langle D\rangle \right)}^{2}}{2{\sigma_{D}}^{2}}\right]$$

where ⟨*D*⟩ is the median diffusion coefficient and *σ* describes the width of the distribution. The scaling law implies that both *P*_LN_(*D*) and *W*_LN_(*M*) are log‐normally distributed as the following applies:

(11) $$\begin{array}{ccc} W_{\mathrm{LN}}\left(M\right) & = & \frac{1}{\sqrt{2\pi }\sigma_{D}AM_{w}^{-\alpha }}\exp\left[\frac{-{\left(\log M-\log\langle M_{w}\rangle \right)}^{2}}{2{\sigma_{M}}^{2}}\right]\\ & & \mathrm{with}\;\log\langle M_{w}\rangle =\frac{\log A-\log\langle D\rangle }{\alpha }\;\;\mathrm{and}\;\sigma_{M}=\frac{\sigma_{D}}{\alpha } \end{array}$$

The subsequent calculation of *M*_w_, *M*_n_, and *Đ* from the MMDs obtained by either the ILT or the log‐normal approach was carried out using Equations (1), (2), (3), where *M*_i_ are the molar masses obtained from Equation 6 and W(*M*_i_) the corresponding probabilities at each point of the distribution.

**Figure 3:**
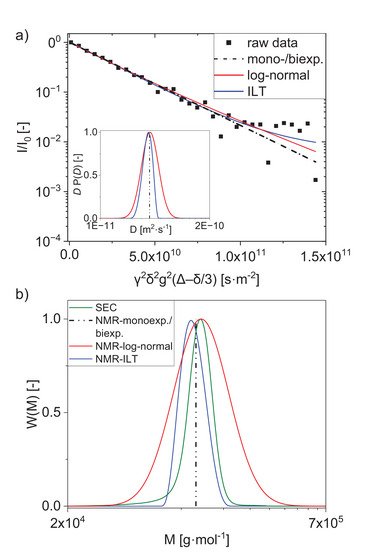
Determination of diffusion coefficients and molar masses for PS123k with PFGSTE‐NMR using different methods. a) Signal attenuation fitted with monoexponential, biexponential, log‐normal, or an inverse Laplace transformation (ILT). The inset shows the diffusion coefficient distributions obtained by the log‐normal model or ILT. *P*(*D*) is shown as *D*·*P*(*D*) to present ⟨*D*⟩ at the maximum of the distribution. b) Corresponding molar mass distributions compared with that from SEC, scaled to the maximum peak height. Diffusion coefficients and molar masses determined through monoexponential and biexponential fits are indicated with straight lines.

Sample PS123k was analyzed with the four methods presented above **1)** the monoexponential fit, **2)** the biexponential fit, **3)** the log‐normal distribution, and **4)** the ILT. The results were compared with data obtained by the SEC. The nearly linear signal decay indicates a narrowly distributed sample, described at low gradient strengths in a very similar way by all four methods (**Figure** **3**a). However, differences in the fitting models can be observed with increasing gradient strength. This results in different DCDs obtained with the log‐normal approach and with the ILT (inset of Figure 3a, where the *D* obtained with the monoexponential or biexponential fit is indicated through a straight line).^[^ ^29^ ^]^ The MMDs for PS123k retrieved from the DCDs determined with the log‐normal model or the ILT were compared to the MMDs from SEC (Figure 3b, with the *M*_w_ obtained using the mono‐ and biexponential fits shown as straight lines). Molar masses and dispersities determined with all 4 methods were compared with values from SEC (**Table** **1**).

**Table 1:** Average molar masses and dispersitiy determined from a PFGSTE‐NMR experiment for anionically synthesized PS123k with different methods: monoexponential fit, biexponential fit, log‐normal distribution, and inverse Laplace transformation (ILT); summary of the advantages and drawbacks of the different methods. Values are compared with reference data obtained with SEC.

Method/fit	*M*_w_ [g·mol^−1^]	*M*_n_ [g·mol^−1^]	*Đ*	Advantages	Drawbacks
SEC	123 000	117 000	1.05	distribution directly accessible from chromatogram, robust and simple data evaluation	frequent recalibration necessary, results depend on column quality
NMR‐monoexp.	117 000	117 000	1.00	simple and fast, numerically stable	no distribution information
NMR‐biexp.	117 000	117 000	1.00	simple and fast, numerically stable, determination of 2 *D*s and weight fractions possible in bimodal samples	limited information about the distribution, especially for narrow distributions numerically ill defined
NMR‐log‐normal	148 000	128 000	1.16	analytically solvable, distributions available	number of components must be known, specific distribution shape is assumed, high standard deviations related to the determination of *Đ* of narrowly dispersed samples
NMR‐ILT	116 000	113 000	1.03	distribution available, no assumption about distribution shape, regularization techniques possible	ill‐posed problem, numerically unstable, and appearance of artifacts possible, dependent on ILT digitization and regularization parameters

Similar values of *D* = 3.85·10^‐11^ m^2^·s^−1^ were obtained for both the **1)** monoexponential and **2)** biexponential fits. The calculated weight fraction, *X*_wt_, of 1.0 for the biexponential fit leads to a negligible value for 1‐*X*_wt_ and thus *D*_2_ (Equation 7), showing that the biexponential fit is not able to resolve different *D*s in a narrowly distributed monomodal sample. Likely, data with higher SNR or samples with very different *D*s would be required to determine two *D*s.^[^ ^36^ ^]^ Overall, using mono‐ or biexponential fittings is simple and robust. With a 5 % deviation from the *M*_w_ SEC value, both are suitable fitting models for calculating average molar masses in uniform, monomodal samples.

**3)** The fit parameters with a log‐normal distribution are reduced to two parameters ⟨*D*⟩ and *σ*_D_, through the assumption that the experimental data follows this shape. Although ⟨*D*⟩ = 3.90·10^‐11^ m^2^·s^−1 ^is close to that from the mono‐ and biexponential fit, *σ*_D_ = 0.234 ± 0.074 shows a coefficient of variation of ≈30 %, leading to a large uncertainty in the calculated dispersity. This results in a much broader MMD than the reference distribution from SEC (Figure 3b), and thus, the calculated molar masses exhibit a great deviation (up to 20 %) from the reference data. High deviations in *σ* can result from not exactly lognormally distributed data. The MMD of PS123k obtained with SEC shows a slightly asymmetric distribution with a tailing toward lower molar masses (Figure 3b). This cannot be considered with the log‐normal model which is only described through the first two statistical moments and the assumption of a Gaussian shape on the log‐scale. In addition, log‐normal distributions are sensitive to outliers or extreme values in the data, which can strongly influence the estimation of the mean and standard deviation. Increasing scatter of the data with increasing gradient strength (Figure 3a) corresponds to low signal intensities *I/I*_0_ and thus noisy data with difficulties in baseline correction. The need for strong spin dephasing in PFG experiments leads to low *I/I*_0_ at higher gradients and is further exacerbated by the need for low polymer concentrations (below c*). The poorer SNR of low‐field NMR compared to high‐field instruments also affects the data quality and possibly the precision of molar mass determination. Although increasing the number of scans could reduce the noise, the method is optimized here for fast analysis and uses only 8 scans per step (see details for method optimization in Section 3.1). Increasing the number of scans or gradient steps would increase the measurement time, making the log‐normal method more time‐consuming than SEC or other mathematical approaches to fitting NMR data such as ILT, where regularization parameters can be used to minimize the effect of noisy data. Identifying small deviations from the ideal, uniform case also results in higher uncertainties regarding the width and shape of the distribution, as well as the statistical moments calculated from it. Although the log‐normal distribution is not optimal for describing narrow or asymmetric distributions, it could be an appropriate model for broad distributions, such as those common for radically synthesized polymers. Figure S8 (Supporting Information) shows the MMD of a bulk polymerized PS sample (*M*_w_ = 133 kg·mol^−1^, *Đ* = 1.53). The log‐normal fit agrees with the SEC molar mass distribution for this broadly distributed sample. A possible reason for the better representation by the log‐normal fit of broadly distributed samples compared to narrowly distributed samples could be that the log‐normal distribution inherently requires a minimum width, and the greater deviation from the ideal monoexponential signal decay makes it easier to estimate a distribution. For commercial samples, which are typically broadly distributed, the log‐normal distribution may therefore be a suitable method, provided there is prior knowledge of the number of components and the sample exhibits a symmetric distribution.

**4)** When using the ILT approach, it is important to note that the ILT is a mathematically ill‐posed problem that impedes its applicability. Even small changes in the input data, such as data acquisition, baseline corrections, and phase corrections, can result in significant changes in the output data. Such numerical instabilities can lead to artifacts. Real signals can be distinguished from artifacts because the latter often have a significantly lower intensity than the former. They usually occur at the distribution edges with diffusion coefficients outside the expected range and show a lack of reproducibility. When the experiment is repeated, they disappear or change position while the true signals remain constant. Overall, the reliability of this method is highly dependent on the quality of the data and the applied mathematical protocol. To obtain more stable and robust solutions even when the NMR data is slightly scattered at increasing gradient strength (Figure 3a), Tikhonov regularization and second derivative smooth were used in this work, which is implemented in the utilized GNAT software.^[^ ^29^ ^]^ With this approach, the number of different solutions can be limited and numerical instabilities can be minimized.^[^ ^37^ ^]^ Additionally, by using appropriate regularization techniques noise in the NMR data can be reduced which improves the stability of the inversion. In theory, ILT does not require any prior knowledge about the distribution shape. However, the reliability of ILT‐generated distributions depends on the chosen regularization and digitization parameters. For comparable results, the utilized ILT algorithm, regularization, and digitization parameters must remain constant.

For the narrowly distributed sample PS123k, similar values of *M*_w_, *M*_n_, and *Đ* could be obtained with deviations of <6 % from the reference SEC data, showing that ILT is a suitable method for the analysis of anionically synthesized polymers with the chosen parameters (Table 1).

For the broadly distributed sample PS133k, it was shown that the log‐normal distribution could be an appropriate fitting model, as discussed previously (Figure S8, Supporting Information). To see if ILT could also be a suitable model for broadly distributed polymers the same sample was analyzed using ILT. The ILT parameters were kept constant except this time a first derivative smoothing was used, as a second derivative smoothing seemed to over‐smooth the data. The result shows a narrower MMD (*Đ* = 1.22) than with the log normal model (*Đ* = 1.52) or the SEC reference (*Đ* = 1.53), however, the mean of the distribution shows good agreement with the other methods (Figure S8, Supporting Information). Overall, this result demonstrates that it is in principle possible to analyze broadly distributed samples with ILT, although the ILT parameters should be further optimized for the analysis of broadly distributed samples. To obtain reliable results with ILT, prior knowledge of the sample (narrowly or broadly distributed) is advisable. In addition, different ILT algorithms may give different results, so prior validation is required when using ILT as a method for molar mass determination (see Section 3.2.3 for narrowly distributed samples). Nevertheless, ILT can represent a wide variety of distribution types, whether narrow, broad, or asymmetric, including those with completely overlapping peaks in the spectral dimension (see Section 3.3.1).

An alternative approach to obtaining distribution information is through dynamic light scattering (DLS). The underlying principle of DLS is based on determining an autocorrelation function from the frequency shift of the scattered light intensity and subsequently calculating *D* from this autocorrelation function. Analogous to NMR experiments, either with an ILT or the cumulant method, the DCDs can be obtained. Similar to NMR, samples must be diluted below c*. Drawbacks of DLS are that it is sensitive to impurities, that no information on the chemical structure is obtained, and that it yields results intensity‐weighted as *I* ∼ *d* ^6^. This can lead to particles with smaller molar masses being obscured by larger particles, especially for heterogeneous samples with small and large molar masses.^[^ ^38^ ^]^ A further drawback of DLS is that the refractive indices of the polymer and the solvent must be different as a prerequisite for the light to scatter. The DLS‐ILT distributions calculated with the calibration parameters derived from NMR are consistent with NMR‐ILT results (Figure S9, Supporting Information), although the number of data points was limited for DLS measurements using the commercial software showing a lower diffusion coefficient resolution compared to NMR‐ILT data.

Overall, the choice between the methods discussed above depends on several factors, including sample type, data quality, and the availability of prior knowledge about the distribution shape. Table 1 summarizes the advantages and drawbacks of the previously discussed methods.

#### 3.2.3 Cross‐Validation of the Optimized Pulsed Field Gradient Stimulated Echo (PFGSTE) Method

To cross‐validate the PFGSTE experiment optimized in Section 3.1, seven monomodal, uniform PS samples with different molar masses were analyzed using a monoexponential fit and ILT. These approaches were chosen due to the advantages and drawbacks of different mathematical models, as discussed in the previous section. The values measured with SEC served as a reference. A deviation of the molar masses obtained with NMR and from the reference values lower than 15 % was chosen as the acceptance criterion, as reproducibility of 10–20 % was measured for PS samples in interlaboratory tests with SEC.^[^ ^39^ ^]^ An overview of the obtained *M*_w_, *M*_n_, and *Đ* is shown in **Table** **2** (see Figures S10—S12, Supporting Information for raw data, DCDs, and MMDs).

**Table 2:** Average molar masses of PS samples obtained with PFGSTE‐NMR using the monoexponential fit or an inverse Laplace transformation (ILT). Relative differences between these and reference values from SEC, rel.diff., are listed in italics on the right of the corresponding NMR values.

Sample	Method	*M*_w_ [g·mol^−1^]	*Rel. Diff. [%]*	*M*_n_ [g·mol^−1^]	*Rel. Diff. [%]*	*Đ*
PS10k	SEC	9 460	*–*	9 220	*–*	1.03
	NMR / monoexp.	9 790	*3.49*	9 790	*6.18*	1.00
	NMR / ILT	9 720	*2.75*	9 624	*4.38*	1.01
PS17k	SEC	17 300	*–*	16 900	*–*	1.03
	NMR / monoexp.	17 000	*−1.73*	17 000	*0.59*	1.00
	NMR / ILT	19 000	*9.83*	18 700	*10.65*	1.02
PS63k	SEC	62 600	*–*	58 500	*–*	1.03
	NMR / monoexp.	61 200	*−2.24*	61 200	*4.62*	1.00
	NMR / ILT	63 700	*1.76*	61 400	*4.96*	1.04
PS123k	SEC	123 000	*–*	117 000	*–*	1.05
	NMR / monoexp.	117 000	*−4.88*	117 000	*0.00*	1.00
	NMR / ILT	116 000	*−5.69*	113 000	*−3.41*	1.03
PS271k	SEC	271 000	*–*	260 000	*–*	1.04
	NMR / monoexp.	244 000	*−9.96*	244 000	*−6.15*	1.00
	NMR / ILT	269 000	*−0.74*	256 000	*−1.54*	1.05
PS516k	SEC	516 000	*–*	451 000	*–*	1.14
	NMR / monoexp.	445 000	*−13.76*	445 000	*−1.33*	1.00
	NMR / ILT	510 000	*−1.16*	473 000	*4.88*	1.08
PS931k	SEC	931 000	*–*	790 000	*–*	1.18
	NMR / monoexp.	938 000	*0.64*	938 000	*18.61*	1.00
	NMR / ILT	1 240 000	*33.19*	985 000	*24.68*	1.26

The NMR data is consistent with the reference data across a broad range of molar masses (10k to 500k) with deviations lower than 14 % for both the ILT and the monoexponential fit. To estimate the reproducibility of the molar mass determination sample PS123k was measured three times on three different days using both NMR and SEC. The coefficient of variation for NMR‐ILT measurements was between 3 and 13 % for *M*_w_, *M*_n_, and *Đ*, showing a reproducibility slightly worse than for SEC measurements (1–7 %) or *M*_w_ calculations with a monoexponential fit (4 %). Discrepancies between the results derived with ILT and the reference data may occur due to the limited number of data points describing the distributions' shape and the fact that performing an ILT is a mathematically ill‐posed problem as previously mentioned.^[^ ^27^ ^]^ For sample PS931k the *M*_w_ determined with ILT deviated from the reference *M*_w_ by ≈33 %, while the deviation for the monoexponential fit was below 1 %. This discrepancy may be attributed to an insufficient signal decay, as PS931k *I*/*I*_0_ decayed only to 0.2, resulting in limited data points for stronger gradients and leading to an inaccurate determination of the distribution. Since the diffusion parameters *Δ* and *δ* have already been maximized for the molar mass range investigated here, as discussed in Section 3.1, stronger field gradients in the spectrometer would be necessary to extend the limits of accurate determination of MMDs toward even higher molar masses. The *M*_w_ calculation using a monoexponential fit is nevertheless sufficiently precise, given that the *M*_w_ calibration was performed with a monoexponential fit, averaging any deviations resulting from a weaker signal decay. Furthermore, the data quality has smaller effects on the calculation with a monoexponential fit as this method is reduced to only one degree of freedom.

### 3.3 Molar Mass Determination for Bimodal Samples and Copolymers

#### 3.3.1 Bimodal Polystyrene (PS) Samples

A challenge in PFG NMR is to differentiate the diffusion coefficients of several polymers whose NMR signals partially or completely overlap. Signal overlap in complex mixtures leading to incomplete spectral resolution is often a problem, especially at low‐fields. For homogeneous, monomodal samples the average diffusion coefficient *D*, extracted with a monoexponential fit is relevant to represent the whole sample. However, for polymers overlapping in their chemical shifts, this approach would yield an intermediate diffusion coefficient between those of the overlapping analytes. The approach of the ILT can resolve two or more *D*s.^[^ ^37^ ^]^ Furthermore, a biexponential fit can model a two‐component polymer solution, each polymer having a certain molar mass and weight fraction.^[^ ^24^, ^36^ ^]^

**Figure 4:**
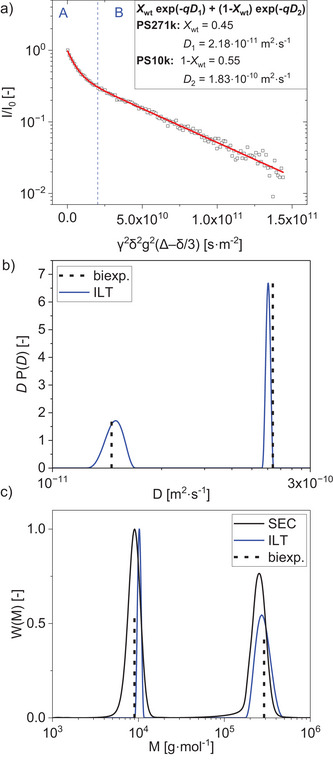
Raw data and distributions obtained with PFGSTE NMR of a bimodal PS271k/PS10k sample with equal weight fractions. a) Stejskal‐Tanner plot with a biexponential fit. The first part (A) represents the faster‐diffusing, smaller molecules while the larger, slower‐diffusing molecules are dominantly represented in the second part (B). b) Diffusion coefficient distribution (DCD) obtained by an inverse Laplace transformation (ILT). c) Molar mass distribution (MMD) calculated from the DCD with calibration parameters for PS in CDCl_3_ and comparison with MMD obtained with SEC. DCDs were scaled to the peak area and MMDs were scaled to the maximum peak height. Diffusion coefficients and molar masses obtained with the biexponential fit are indicated with dotted lines. See Figures S13–S15 (Supporting Information) for the corresponding plots for other polymer blends of Table 3.

The potential to measure the distinct *D*s of a mixture of homopolymers of the same chemical nature with a low‐field NMR spectrometer was assessed by measuring blends of two PS samples with different molar masses in different weight fractions (**Table** **3**). Both a biexponential fit and the ILT were used to determine *M*_w_ values and weight fractions. Additionally, SEC measurements were conducted for comparison. The Stejskal‐Tanner log‐plot of a blend of PS271k/PS10k with equal weight fractions clearly displays a superposition of two exponential decays (**Figure** **4**a). The first part of the curve (A) shows a steeper signal decay caused by the smaller and faster diffusing molecules (PS10k), while the second part (B) shows the decay of the slower diffusing, higher molar mass PS271k. The spectra were recorded using the same parameters as described in Section 3.1, except for the step number which was increased to 128 to obtain a sufficient number of NMR data points. This is necessary for the accurate calculation of the diffusion coefficient of the faster decaying molecule, which is represented in the shorter part A of the signal decay. The DCD and MMD also could be determined by using the ILT. When two separate peaks with similar areas are plotted on a semi‐logarithmic graph, the peaks at larger *D*s show smaller peak areas and vice versa. As described in the literature *P*(*D*) d*D *= *P*(*D*)*D* d(ln*D*) and therefore *D*·*P*(*D*) is plotted.^[^ ^27^, ^37^ ^]^ The DCDs were scaled to the integrals while the MMDs were scaled to the maximum peak height and compared with the MMDs obtained with SEC (see DCDs and MMDs in Figure 4b,c for PS271k/PS10k with equal weight fractions, and Figures S14 and S15, Supporting Information, for other samples). Table 3 shows the results from SEC and NMR measurements, compared to the *M*_w_ of the related monomodal samples.

**Table 3:** Molar masses (in kg·mol^−1^) and relative weight fractions (in wt.%) of bimodal PS samples measured with SEC or with NMR and calculated with either inverse Laplace transformation (ILT) or a biexponential fit. Reference values of weight fractions as prepared and molar masses of monomodal components are shown for comparison. Relative differences between bimodal sample experiment values and monomodal values are listed in Table S5 (Supporting Information).

Sample name	Monomodal components		Bimodal samples, blends of 2 monomodal components					
			SEC		ILT		biexp. fit	
	*M*_w_	*X*_wt_ ^a)^	*M*_wi_	*X*_wt_ ^b)^	*M*_wi_	*X*_wt_ ^b)^	*M*_wi_	*X*_wt_ ^b)^
PS271k/PS10k	10	50	9 252	53 47	10 295	49 51	9 290	55 45
	271	50						
PS271k/PS10k	10	30	10 256	34 66	8 276	29 71	8 252	37 63
	271	70						
PS271k/PS10k	10	70	10 276	71 29	10 226	65 35	9 208	72 28
	271	30						
PS63k/PS17k	17	50	17 63	52 48	18 72	54 46	17 67	58 42
	63	50						
PS17k/PS10k	10	50	9 18	45 55	6 15	92 8	13 19	96 4
	17	50						

^a)^ wt. fraction as prepared;

^b)^ wt. fraction as obtained from measurement/calculation.

Diffusion coefficients obtained with NMR were determined with either a biexponential fit (Equation 7) or the ILT (Equation 8). The molar mass calculations were then carried out using the calibration parameters for PS in CDCl_3_ displayed in Figure 2. For the polymer blends PS271k/PS10k and PS63k/PS17k with equal weight fractions, *M*_w_ values determined by all three methods agree with the *M*_w_ values of the corresponding monomodal samples (within 14 %). In blends with unequal weight fractions, *M*_w_ values obtained with NMR were determined with a larger deviation for the minor component (*X*_wt_ = 0.3, up to 23 %) than for the major component (within 10 %). The calculated weight fractions, *X*_wt_, were obtained from the integrals of the DCDs derived with the ILT and from the amplitudes of the biexponential fit. Both were consistent with the weight fractions as prepared, with smaller deviations for the ILT (within 17 %) than for the biexponential fit of the experimental data (up to 23 %). Overall, the SEC results indicate smaller deviations (<10 %) due to the nearly complete resolution of peaks on the chromatography axis, making it easier to calculate different molar masses of bimodal samples compared to NMR. The comparison of the MMDs of the SEC and NMR‐ILT measurements shows slight differences in the determined width of the distributions. This is due to the substantial increase of parameters to be determined with ILT. The width and shape of the ILT‐distribution must be determined based on small deviations from the monoexponential behavior. Furthermore, as described in the previous sections, the ILT is an ill‐posed problem. This is amplified with an increasing number of components and thus parameters, resulting in higher uncertainties.

To test the limits of PFG NMR using a low‐field NMR spectrometer, it was examined how close the diffusion coefficients, and therefore the molar masses of two homopolymers could get until the determination of each component in their blend was no longer possible with sufficient precision. For a blend of PS17k/PS10k in equal weight fractions (50:50) two distinct populations could still be detected but an error of up to 40 % was made on the determination of the related *M*_w_ values with both methods (Table 3). The weight fractions could not be correctly determined, as both methods strongly underestimated the weight fraction of the slower diffusing, larger molecule (by a factor of >6). The number of scans was increased from 8 to 64 per increment to increase the SNR, however, no further improvement in the relative fraction of the molar mass determination was observed.

Overall, a difference ≈3x in *M*_w_ is necessary to distinguish and quantify both the molar masses and the weight fractions of two components in bimodal samples for the here described methods.

#### 3.3.2 Block Copolymers of PS and poly(methyl methacrylate), PS‐*b*‐PMMA

To distinguish between a copolymer and a blend of the corresponding homopolymers, or a blend of the copolymer and corresponding homopolymers, a simple ^1^H NMR spectrum is not sufficient, except in the case of end‐group analysis of low molar mass polymers. However, PFG NMR can discriminate between the diffusion coefficient of a homopolymer and that of a block of the same length present in a larger copolymer, for which both blocks will exhibit equal and lower diffusion coefficients. Furthermore, the DCD and apparent MMD for each block, as well as the chemical composition profile for the block copolymer, can be determined using for example the ILT.^[^ ^16^ ^]^ Four PS‐*b*‐PMMA block copolymers with different compositions and molar masses (**Table** **4**) were characterized using the 90 MHz benchtop NMR spectrometer and the results were compared with already published data obtained at high field (700 MHz) on the exact same samples.^[^ ^16^ ^]^ An additional calibration for PMMA was performed for molar mass determinations, which exhibited a linear correlation similar to that of the PS calibration curve (Figure 2). To determine the DCD for each block, the signals of the aromatic protons of PS and the methoxy group of PMMA were integrated, plotted against the gradient strength (Figure S16, Supporting Information), and then the ILT was performed (Figure S17, Supporting Information). Additionally, it is possible to calculate the evolution of the chemical composition as weight fractions (*w*, wt.%) of PS and PMMA, which can vary significantly across the DCD and thus the MMD. This is determined through the ratios of the intensities of the aromatic protons of PS and the methoxy group of PMMA (*I*_PS_ and *I*_PMMA_) at a given *D*:

(12) $$\begin{array}{c} w_{\mathrm{PS}}\left(D\right)=\frac{\frac{I_{\mathrm{PS}}\left(D\right)\cdot M_{S}}{n_{\mathrm{PS}}}}{\frac{I_{\mathrm{PS}}\left(D\right)\cdot M_{S}}{n_{\mathrm{PS}}}+\frac{I_{\mathrm{PMMA}}\left(D\right)\cdot M_{\mathrm{MMA}}}{n_{\mathrm{PMMA}}}}\;\text{and}\;w_{\mathrm{PMMA}}\left(D\right)=1-w_{\mathrm{PS}} \end{array}$$

where *n*_PX_ is the number of protons in the integrated signal of polymer PX and M_X_ is the molar mass of its monomer unit.

**Table 4:** Comparison of average molar masses (in kg·mol^−1^) and dispersity of PS‐*b*‐PMMA copolymers obtained at low field (90 MHz, this work) and published on the exact same samples at high field (700 MHz),^[^ ^16^ ^]^ both calculated with inverse Laplace transformation. PS‐ or PMMA‐equivalent molar masses were determined independently for “true” copolymer molar masses. Average molar masses supplied by the manufacturer (SEC) are also given for comparison.

*M*_w_ reference (SEC)	NMR ^1^H Larmor frequency	Copolymer			PS‐equivalent			PMMA‐equivalent		
		*M*_w_	*M*_n_	*Đ*	*M*_w_	*M*_n_	*Đ*	*M*_w_	*M*_n_	*Đ*
50	700 MHz	45	42	1.05	42	40	1.06	47	45	1.03
	90 MHz	49	48	1.02	48	46	1.03	51	50	1.02
81	700 MHz	89	81	1.10	86	75	1.15	92	86	1.07
	90 MHz	93	90	1.04	89	84	1.06	96	93	1.03
84	700 MHz	85	79	1.07	83	77	1.07	88	83	1.06
	90 MHz	91	88	1.03	82	80	1.02	100	97	1.03
108	700 MHz	102	94	1.08	105	97	1.08	100	91	1.10
	90 MHz	104	100	1.04	100	97	1.03	107	101	1.06

For the determination of the MMD of either the PS‐ or the PMMA‐equivalent molar masses of the copolymer, Equations 6 and 9 were used with the parameters of the calibration curves for PS or PMMA in CDCl_3_, respectively. The PS‐ and the PMMA‐equivalent *M*_w_, *M*_n,_ and *Đ* were then calculated with Equations (1), (2), (3).

The “true” molar mass of the copolymer *M*_cp_(*D*) at each diffusion coefficient was then calculated by adding the PS‐ and the PMMA‐equivalent molar masses (*M*_PS_(*D*) and *M*_PMMA_(*D*)) weighted by their weight fractions:

(13) $$M_{\mathrm{cp}}\left(D\right)=w_{\mathrm{PS}}\;\left(D\right)M_{\mathrm{PS}}\left(D\right)+w_{\mathrm{PMMA}}\left(D\right)M_{\mathrm{PMMA}}\left(D\right)$$

**Figure 5:**
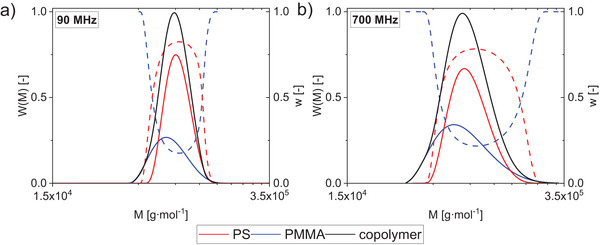
Molar mass distribution (*W*(*M*), solid lines) and chemical composition evolution (*w*, dotted lines) of PS‐*b*‐PMMA81k obtained with a) a 90 MHz NMR spectrometer or b) a 700 MHz NMR spectrometer. Data at 700 MHz NMR is from Bastian Grabe and Wolf Hiller, Macromolecules 2022, 55, 8014‐8020, https://doi.org/10.1021/acs.macromol.2c01505.

*P*(*D*) was converted into the mass distribution *W*(*M*) using Equation 6 with the calibration parameters for PMMA, the major component of the copolymers. The calculation for *M*_w_ and *M*_n_ was then performed similarly to Equations 1 and 2, except that *M*_i_ is the composition weighted molar masses *M*_cp_(*D*) obtained from Equation 13. **Figure** **5**a shows for sample PS‐*b*‐PMMA81k the evolution of the chemical composition (PS and PMMA) as a function of the molar mass as well as the MMD of the copolymer obtained with the 90 MHz NMR, other samples are shown in Figure S18 (Supporting Information). Figure 5b shows for the same sample the results obtained on a 700 MHz NMR spectrometer (data already published^[^ ^16^ ^]^). The determined molar masses are gathered for all samples in Table 4.

The 90 and 700 MHz NMR spectrometers yielded consistent results. The experiments on both instruments show that the copolymer's PS‐ or PMMA‐equivalent molar masses differ slightly. For PS‐*b*‐PMMA 50k and 81k, PS exhibits a broader MMD shifted to lower molar masses. A possible explanation could be the presence of a minor component, such as a PS homopolymer,^[^ ^16^, ^24^ ^]^ which could be produced via oxidative coupling of anions in a side reaction, as no other peaks of other substances were identified in the spectra. Additionally, the chemical composition of the copolymers can be analyzed. Similar results to the data at the high‐field were observed, where the intensity of PS‐*b*‐PMMA 50k and 81k is highest in the lower molar mass regions, then decreases and is highest again in the highest molar mass regions.^[^ ^16^ ^]^ The results with *w* = 0.0 or 1.0 at low and high molar masses of the distributions should be considered with caution, as weak intensities of the DCDs/MMDs cause greater uncertainties. In addition, the copolymers were analyzed in this work using SEC with UV and DRI detection. The comparison of the copolymer MMDs obtained with NMR‐ILT at high field,^[^ ^16^ ^]^ NMR‐ILT at 90 MHz, and SEC all exhibit similar shapes (see Figure S19, Supporting Information). In contrast to the copolymer MMDs obtained by NMR‐ILT, the MMDs obtained from SEC‐DRI/UV analysis exhibit an additional small, overlapping peak toward lower molar masses for the block copolymers 50k and 81k. This could be another indication of residual homopolymers. The intensity of the additional small peak is greater in the UV chromatogram than in the DRI detector after their normalization to the main peak maximum (Figure S20, Supporting Information). As at 254 nm, there is only PS absorption and negligible PMMA absorption, the SEC measurements confirm that those samples contain relatively more PS than PMMA at lower molar masses.^[^ ^40^ ^]^ Further information on the chemical composition evolution of copolymers could be obtained by performing SEC‐NMR measurements at low fields.^[^ ^41^, ^42^, ^43^ ^]^ For PS‐*b*‐PMMA108k the PS‐equivalent molar mass of the copolymer was again slightly lower than the PMMA‐equivalent one, contrary to what was observed at high‐field (Table 4). This difference could be attributed to the use of different calibration curves. However, the chemical composition distribution shows the same trend, with relatively more PMMA in the highest and lowest molar mass fractions for PS‐*b*‐PMMA108k, which is consistent with the broader dispersity of the PMMA fraction.

### 3.4 Diffusion Measurements in Protonated Solvents

**Figure 6:**
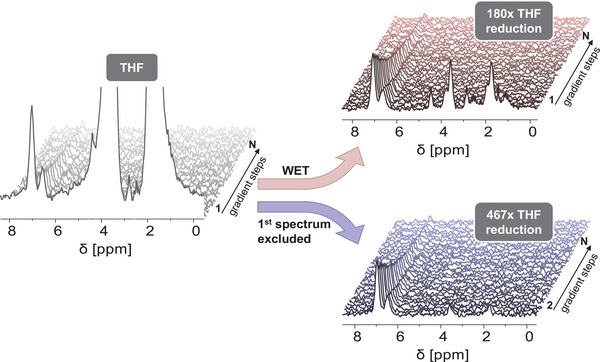
^1^H‐PFGSTE NMR of PS516k in THF with *N* = 32 gradient steps, without solvent suppression (left), with WET suppression of protonated THF signals at 3.6 and 1.7 ppm, leading to a THF signal reduction by a factor of 180 (top right) and excluding the first spectrum (gradient step number 1), leading to a THF signal reduction by a factor of 467 (bottom right). Aliphatic protons of the PS backbone are not visible due to a *T*_1_ relaxation time (≈100 ms) shorter than the diffusion delay of 300 ms.

Benchtop NMR spectrometers can record spectra in protonated solvents without deuterated solvents due to an external ^19^F‐lock. This eliminates the need for more expensive deuterated solvents.^[^ ^18^ ^]^ However, non‐deuterated solvents such as THF lead to intense and broad signals as the sample contains >99 wt.% solvent (**Figure** **6**). It is possible to integrate the PS aromatic signals after precise phase correction and an appropriate local baseline correction. However, the much larger THF signals significantly affect those corrections and thus the signal intensities of the analytes, resulting in greater deviations in the determined *D*s.

To minimize solvent signals, one option is to use solvent suppression techniques before data acquisition using specific pulse sequences, such as the presaturation method or WET suppression (water suppression enhanced through the *T*_1_ effect).^[^ ^18^, ^19^ ^]^ In this work WET suppression was applied, a pulse sequence with solvent‐selective radio frequency pulses, followed by dephasing gradient pulses. To avoid any partial suppression of analyte signals, the suppressor frequencies were selected to be precisely those of the two THF signals at 3.58 and 1.73 ppm. This effectively reduced the solvent signals in the first spectrum by a factor of 180 (Figure 6). The resulting spectral resolution of the PS aromatic proton signals from the THF signals allows a more accurate integration of these analyte signals and an efficient use of the dynamic range of the analog‐to‐digital converter.

An alternative way of suppressing the solvent signal in PFG NMR experiments could be to exclude the first spectrum (gradient step no. 1) and normalize everything to the intensity of the second spectrum since the signal intensity of rapidly diffusing small solvent molecules will have already reduced to nearly 0 at the second gradient step (Figure 6). Here a THF suppression from the first to the second gradient step by a factor of 467 could be observed.

To assess the accuracy of molar mass determination in protonated solvents either using WET‐PFGSTE or by eliminating the first data point of an experiment without solvent suppression, a calibration of PS in THF was performed (Figure 2). The WET‐PFGSTE calibration curve shows a linear correlation (R^2^ higher than 0.99) over a range from 1 to 1000 kg·mol^−1^. Furthermore, the value of *α* = 0.61 suggests that THF is a suitable solvent for PS and is in good agreement with the value of *α *= 0.62 reported previously.^[^ ^34^ ^]^ Sample PS516k was measured in protonated THF with and without solvent suppression and the mean *D* was calculated with a monoexponential fit (Figure S21, Supporting Information). The results show that both methods led to very similar *D*s, 1.81 × 10^‐11^ and 1.78 × 10^‐11^ m^2^·s^−1^, resulting in calculated *M*_w_ of 510 and 521 kg·mol^−1^.

This indicates that measuring samples in protonated THF is possible, without affecting the analyte signals due to solvent suppression or equivalently by excluding the first spectrum at the first gradient step.

## 4 Conclusion

A pulsed field gradient method for measuring diffusion coefficients and obtaining molar masses on a 90 MHz benchtop NMR spectrometer with a gradient strength of ≈0.5 T·m^−1^ was optimized and evaluated on polymer model systems. The optimized diffusion measurement for monomodal polystyrene samples took ≈11 min, using 32 gradient steps, 8 scans per spectrum, a gradient pulse duration of *δ* = 5 ms, and a diffusion delay time of *Δ* = 300 ms, making it a time‐efficient method. A simple sample preparation, with 2.5 g·L^−1^ polymer concentration and small solvent amounts (<1 mL), supports this method. Beyond the determination of a mean diffusion coefficient and of the *M*_w_ from it with a monoexponential or biexponential fit, diffusion coefficient distributions (DCDs) and molar mass distributions (MMDs) can be obtained using the log‐normal model or the inverse Laplace transformation (ILT). The DCDs and MMDs determined by log‐normal and the ILT were compared, whereby the ILT presented the shape and width of the distribution slightly better for narrowly distributed samples. Even though ILT is an ill‐posed problem with drawbacks, it has been demonstrated that ILT is a valuable method for obtaining information about MMDs with a benchtop spectrometer. Additionally, the ILT allows for the extraction of diffusion coefficients of bimodal samples with two spectrally overlapping signals, enabling the calculation of their DCDs, MMDs, and weight fractions. Similar results could be obtained for the average molar masses and weight fractions by processing the data with a biexponential fit. The MMDs and evolution of chemical compositions as a function of molar masses of block copolymers determined here with low field‐NMR (90 MHz) was consistent with published data from high‐field NMR (700 MHz) on the same samples. Furthermore, it has been shown that it is possible to measure polymer samples even in protonated solvents using a benchtop spectrometer. By combining WET solvent suppression (water suppression enhanced through the *T*_1_ effect) with the pulsed field gradient stimulated echo (PFGSTE) pulse sequence solvent peaks were suppressed by a factor of 180. In addition, protonated THF was shown to be reduced by a factor of ≈470 from the first to the second gradient step using the here‐optimized PFGSTE method. Similar molar masses could be determined either by using WET or by simply excluding the first spectrum of the PFGSTE experiment.

Despite the lower gradient field strengths, a lower SNR, and poorer spectral resolution, it is possible to obtain DCDs and MMDs using a low‐field NMR spectrometer. Overall, the method demonstrates valid results for monomodal and bimodal PS samples as well as for PS‐*b*‐PMMA block copolymers, verified by SEC or high‐field NMR. It offers a simpler, more cost‐effective, and space‐saving alternative to high‐field spectrometers, opening promising perspectives for the use of benchtop spectrometers in the future.

## 5 Experimental Section

### Materials

For molar mass determination polystyrene (PS) samples were either synthesized in‐house by anionic polymerization or purchased from Polymer Standards Service GmbH (PSS, Mainz, Germany). Corresponding weight‐average molar mass *M*_w_ and dispersity *Ð* were characterized by SEC, see **Table** **5**. To calibrate the NMR and SEC systems, additional standards of PS and poly(methyl methacrylate), PMMA, were also obtained from PSS (see Table S1 in Supporting Information for their molar masses). PS‐*b*‐PMMA block copolymer samples, see Table 5, were kindly provided by the group of Prof. W. Hiller, TU Dortmund, Germany.^[^ ^16^ ^]^ The polymer samples were dissolved in deuterated chloroform (CDCl_3_, 99.8 % with 1 v/v % TMS, Thermo Fisher Scientific, USA) or tetrahydrofuran (THF, GPC grade with butylated hydroxytoluene (BHT), Thermo Fisher Scientific, USA).

**Table 5:** Weight‐average molar mass (*M*_w_) and dispersity (*Đ*) of the investigated samples, determined by SEC.

Sample name	Source	*M*_w_ [kg·mol^−1^]	*Đ*
PS10k	in‐house	10	1.03
PS17k	commercial	17	1.03
PS63k	commercial	63	1.03
PS123k	in‐house	123	1.05
PS271k	commercial	271	1.04
PS516k	commercial	516	1.14
PS931k	commercial	931	1.18
PS‐*b*‐PMMA50k	Hiller^[^ ^16^ ^]^	50	1.07
PS‐*b*‐PMMA81k	Hiller^[^ ^16^ ^]^	81	1.11
PS‐*b*‐PMMA84k	Hiller^[^ ^16^ ^]^	84	1.07
PS‐*b*‐PMMA108k	Hiller^[^ ^16^ ^]^	108	1.08

### SEC Analysis

Molar masses and dispersities of all samples were determined using an SEC system (Agilent 1200 series), equipped with a refractive index detector (35 °C) and a UV detector (1200 series). THF was used as a solvent and mobile phase at 1 mL·min^−1^, and 100 µL of each polymer solution at 1 g·L^−1^ was injected. The SEC columns (SDV‐Lux‐1000 Å and SDV‐Lux‐10^5^ Å, from PSS) were calibrated using nine linear PS standards over a *M*_w_ range of 0.7 to 1530 kg·mol^−1^ using a 5th order polynomial fit (R^2 ^> 0.9999) with the software WinGPC UniChrom (see Table S1 and Figure S1, Supporting Information). A conventional calibration with standards of the same chemical nature yielded true molar masses for PS samples.^[^ ^28^ ^]^

### NMR Analysis

The PFGSTE experiments were performed on a Spinsolve 90 Carbon ULTRA benchtop NMR spectrometer (Magritek, Aachen, Germany) operating at a ^1^H Larmor frequency of 90 MHz, with a maximum gradient strength of 0.53 T·m^−1^ and the pulsed field gradients stimulated echo (PFGSTE) pulse sequence (Figure 1). The magnet temperature was set by Magritek to 26.5 °C, and the samples were equilibrated in the magnet for five minutes before measurement. The parameter optimization for diffusion measurements was discussed in detail in Section 3.1. Optimized parameters were as follows: 32 gradient steps with a maximum gradient strength of 98 % (0.52 T⋅m^−1^), *δ* of 5 ms, *∆* of 300 ms, and 8 scans per step. A repetition time of 2.5 s and an acquisition time of 0.8 s were applied, resulting in a measurement duration of ≈11 min. For bimodal samples and samples with molar masses lower than 20 kg⋅mol^−1^, 128 gradient steps and for WET‐PFGSTE a 4 s repetition delay was used. Samples were dissolved in CDCl_3_ or protonated THF (2.5 g·L^−1^) and measured in 5 mm standard NMR tubes (Deutero GmbH, Germany). The spectra were recorded with the Spinsolve (2.3.3) or SpinsolveExpert (1.41.16) software and processed using Mestrelab Mnova (14.1.2) or the GNAT Toolbox (1.3.2).^[^ ^29^ ^]^ NMR spectra were phase corrected, zero‐filled by a factor of 2 and the FID multiplied with an optimized exponential apodization filter of 2 Hz line broadening. Local baseline correction was done with a polynomial fit 1^st^ order. The spectra were integrated into the range of the chemical shift of the aromatic protons of PS (6.3–7.2 ppm) or the methoxy group of PMMA (3.4–3.8 ppm). Diffusion coefficients were obtained by 4 methods from the decay of the normalized signal intensities against the increasing gradient strength: 1) fit to a monoexponential decay (Equation 5), 2) fit to a biexpoential decay (equation 7), 3) fit to a log‐normal model (equation 10), 4) calculated via an ILT (equation 8). The ILT was performed by using the GNAT Toolbox,^[^ ^29^ ^]^ with the following parameters: CONTIN approach, constraint nonnegative, Tikhonov regularisation, smooth 2nd derivative, optimal λ: L‐curve.

Longitudinal relaxation times (*T*_1_) were determined for the aromatic protons of PS3k and PS1200k with an inversion recovery experiment to quantify the expected *T*_1_ values depending on molar masses. With 20 spectra, 4 scans per spectrum, a repetition time of 3 s, and a maximum delay time, *τ*, of 1 s.

## Conflict of Interest

The authors declare no conflict of interest.

## Supporting information

Supporting Information

## Data Availability

Data supporting the findings of this study are available in the supporting information of this article.
