# Supplementary material for: Potential of Benchtop NMR for the Determination of Polymer Molar Masses, Molar Mass Distributions, and Chemical Composition Profiles by Means of Diffusion‐Ordered Spectroscopy, DOSY
Source: Macromol Rapid Commun. 2024 Aug 6;45(24):2400512. doi: 10.1002/marc.202400512 (PMC11661663; doi:10.1002/marc.202400512)
Supplement: Supplementary file 1 — Supporting Information [file MARC-45-2400512-s001.pdf]

Macromolecular Rapid Communications

Supporting Information

for *Macromol. Rapid Commun.*, DOI 10.1002/marc.202400512

Potential of Benchtop NMR for the Determination of Polymer Molar Masses, Molar Mass Distributions, and Chemical Composition Profiles by Means of Diffusion-Ordered Spectroscopy, DOSY

*Johanna Tratz, Marianne Gaborieau, Markus Matz, Michael Pollard and Manfred Wilhelm**

Supporting Information

Potential of Benchtop NMR for the Determination of Polymer Molar Masses, Molar Mass Distributions, and Chemical Composition Profiles by means of Diffusion-Ordered Spectroscopy, DOSY

*Johanna Tratz, Marianne Gaborieau, Markus Matz, Michael Pollard, Manfred Wilhelm**

1. Calibration 2
2. Optimization of experimental parameters 3
3. Broadly distributed polystyrene sample 9
4. Dynamic light scattering (DLS) 10
5. Characterisation of monomodal uniform PS samples 11
6. Characterisation of bimodal PS samples 14
7. Characterisation of PS-b-PMMA block copolymers 18
8. Characterisation of PS in non-deuterated THF 23

1. Calibration

Table S 1 Average molar masses and dispersities of the PS and PMMA standards for NMR and SEC calibration (provided by manufacturer).

PS standards for SEC calibration			PS standards for NMR calibration			PMMA standards for NMR calibration		
M_w [g·mol^{-1}]	M_n [g·mol^{-1}]	*Đ*	M_w [g·mol^{-1}]	M_n [g·mol^{-1}]	*Đ*	M_w [g·mol^{-1}]	M_n [g·mol^{-1}]	*Đ*
690	630	1.10	-	-	-	6 270	5 880	1.07
1 470	1 390	1.06	-	-	-	12 500	12 100	1.03
3 460	3 260	1.06	3 460	3 260	1.06	40 300	38 100	1.06
8 900	8 650	1.03	8 900	8 650	1.03	86 300	82 700	1.04
-	-	-	34 000	32 700	1.04	380 000	372 000	1.02
58 900	53 800	1.09	58 900	53 800	1.09	1 100 000	1 010 000	1.08
125 000	120 000	1.04	125 000	120 000	1.04	-	-	-
552 000	537 0000	1.03	552 000	537 000	1.03	-	-	-
864 000	758 000	1.14	864 000	758 000	1.14	-	-	-
-	-	-	1 170 000	1 070 000	1.09	-	-	-
1 530 000	1 350 000	1.13	-	-	-			

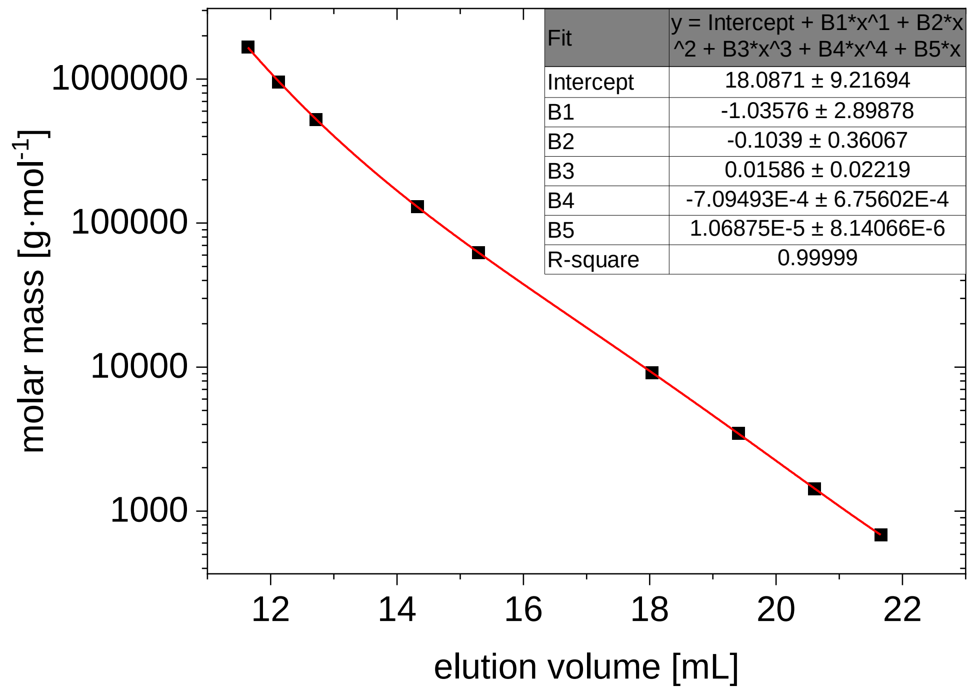

Figure S 1 SEC calibration of PS with 10 linear PS standards in a range of 0.7 to 1530 kg·mol^{-1}, using a 5th order polynomial fit (R^2>0.9999).

2. Optimization of experimental parameters

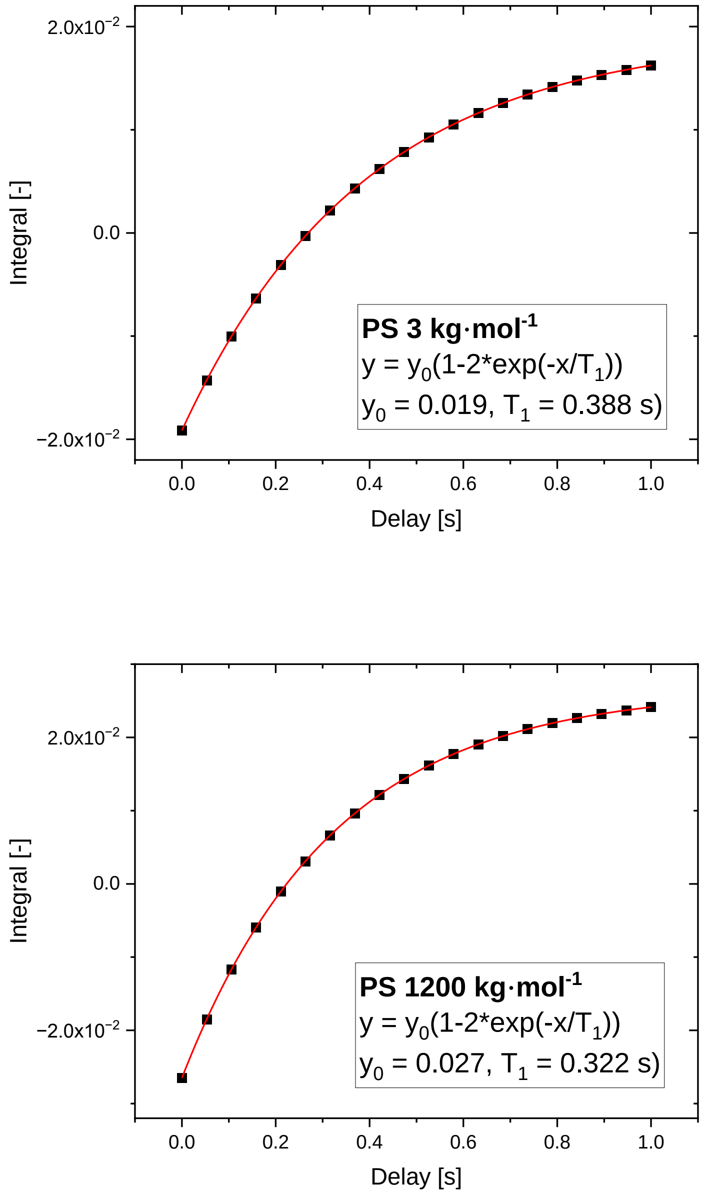

Figure S 2 T_1 measurements for PS3k (top) and PS1200k (bottom) via inversion recovery.

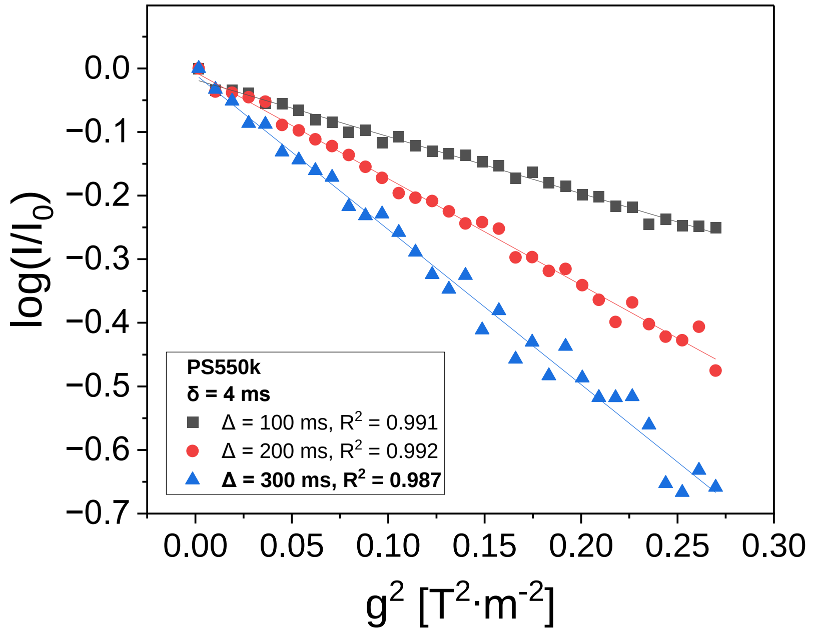

Figure S 3 Signal attenuation of PS550k for various diffusion times, Δ. Gradient pulse length δ was kept constant (4 ms). I/I_0 was plotted on a logarithmic scale to measure the quality of the linear regression, R^2.

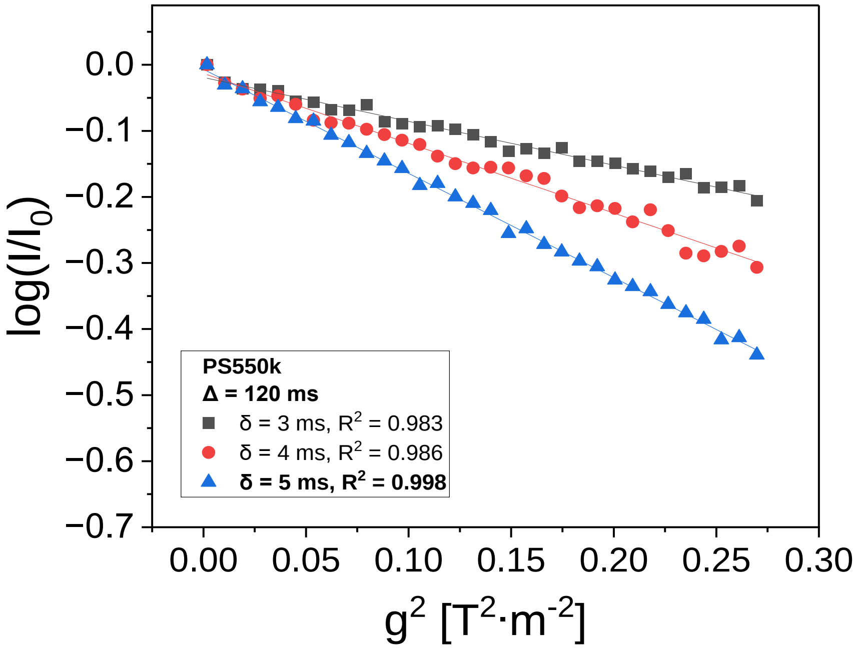

Figure S 4 Signal attenuation of PS550k at varying gradient pulse lengths, δ. Diffusion time Δ was kept constant (120 ms). I/I_0 was plotted on a logarithmic scale to measure the quality of the linear regression, R^2

Table S 2 Optimisation of the number of steps for diffusion measurements (PS550k). 32 steps were used for the optimized method.

	16 steps	32 steps	64 steps
D [$m^2 \cdot s^{-1}$]	1.639E-11	1.637E-11	1.661E-11
	1.531E-11	1.625E-11	1.639E-11
	1.581E-11	1.591E-11	1.655E-11
	1.623E-11	1.580E-11	1.679E-11
	1.558E-11	1.656E-11	1.692E-11
	1.656E-11	1.613E-11	1.630E-11
	1.613E-11	1.628E-11	1.669E-11
	1.697E-11	1.610E-11	1.632E-11
	1.572E-11	1.627E-11	1.673E-11
	1.635E-11	1.619E-11	1.683E-11
$\bar{X}$ [$m^2 \cdot s^{-1}$]	1.61E-11	1.62E-11	1.66E-11
σ [$m^2 \cdot s^{-1}$]	5.01E-13	2.18E-13	2.19E-13
**σ [%]**	**3.11**	**1.35**	**1.32**

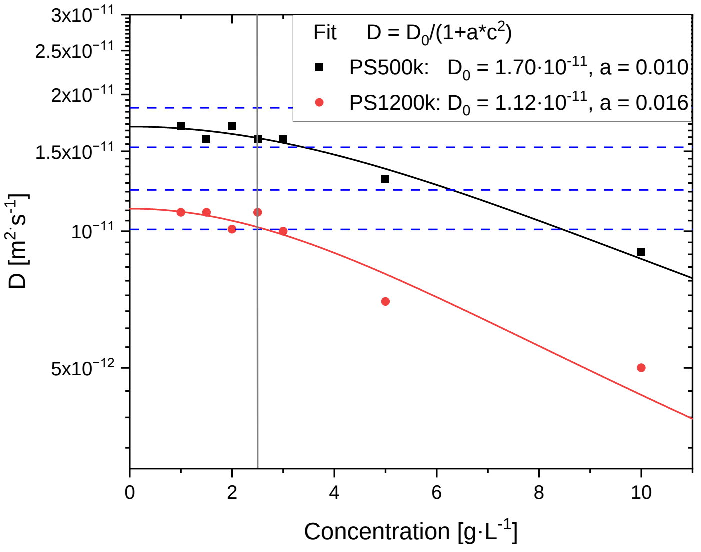

Figure S 5 Diffusion coefficients (D) of PS550k and PS1200k as a function of concentration (c). Horizontal dashed lines indicate a deviation of ±10 % of D at low concentrations. Vertical line at 2.5 g·L^{-1} shows the concentration used for the measurements.

Table S 3 Standard deviation (σ), relative standard deviation (RSD) and signal to noise ratio (SNR) for different numbers of scans (PS550k). 8 scans were used for the optimized method.

Number of scans	<*D*> [$m^2 \cdot s^{-1}$]	σ [$m^2 \cdot s^{-1}$]	RSD [%]	SNR (7.04 ppm)	Measurement time [min]
**2**	1.682E-11	1.057E-12	6.28	18.82	**2.49**
**4**	1.662E-11	4.425E-13	2.66	31.26	**5.39**
**8**	1.608E-11	2.710E-13	1.69	45.18	**11.19**
**16**	1.541E-11	2.361E-13	1.53	59.34	**22.39**
**32**	1.544E-11	2.031E-13	1.32	89.36	**45.19**

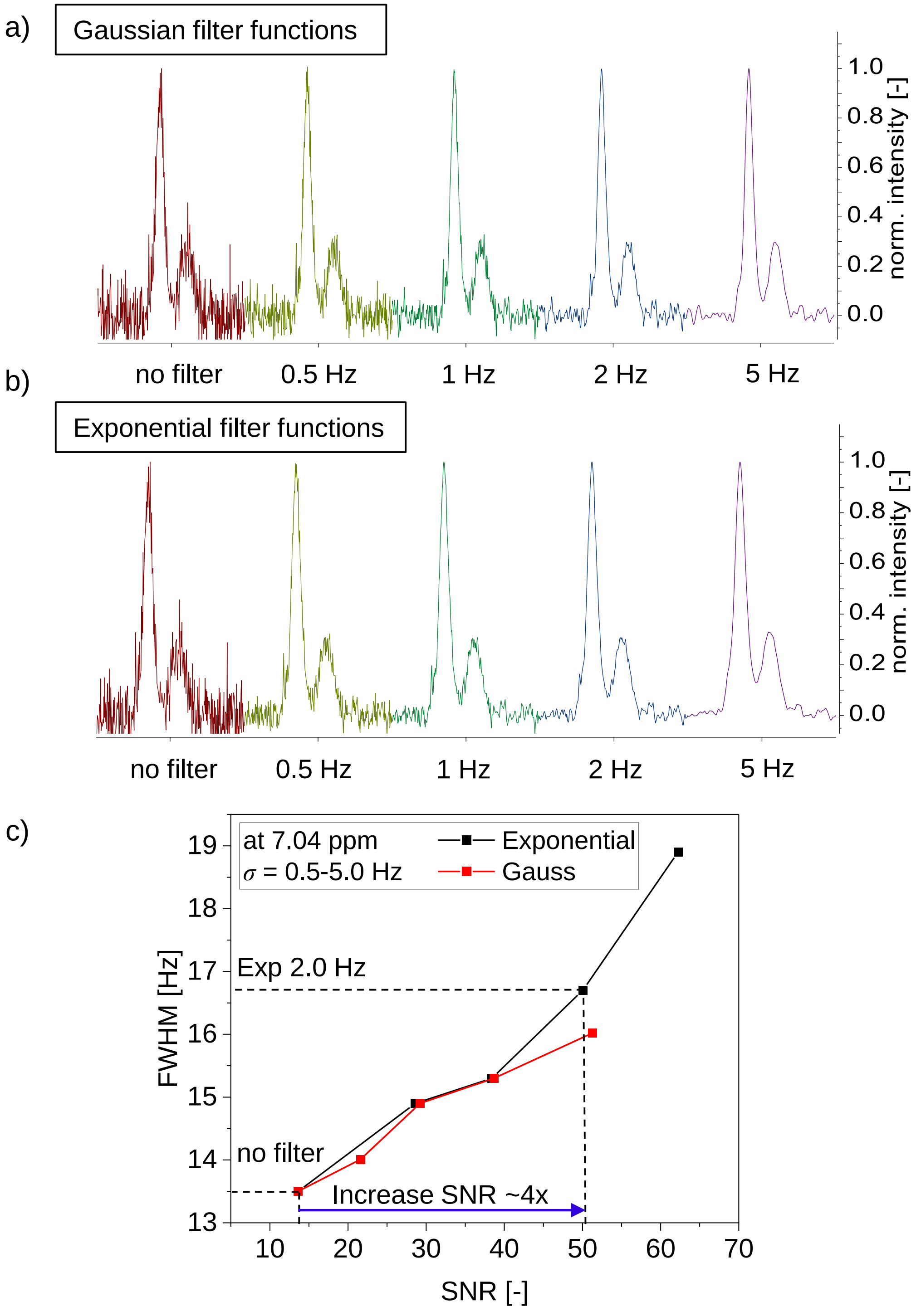

Figure S 6 Comparison of the effects of no, exponential, or Gaussian filter functions with different standard deviations of the peak width (σ): 0.5, 1.0, 2.0, 5.0 Hz on the aromatic protons of PS550k (6.3-7.2 ppm) and the chloroform peak at 7.25 ppm. a) Spectra at the first gradient step of a PFGSTE experiment with different Gaussian filters normalized to a maximum peak intensity of 1. b) Spectra with different exponential filters, c) Related full width at half maximum (FWHM) and signal-to-noise ratios (SNR) from the NMR data. Dotted lines indicate the FWHM and SNR with no filter function and with an exponential filter with σ = 2.0 Hz, leading to an increase in SNR of nearly factor four.

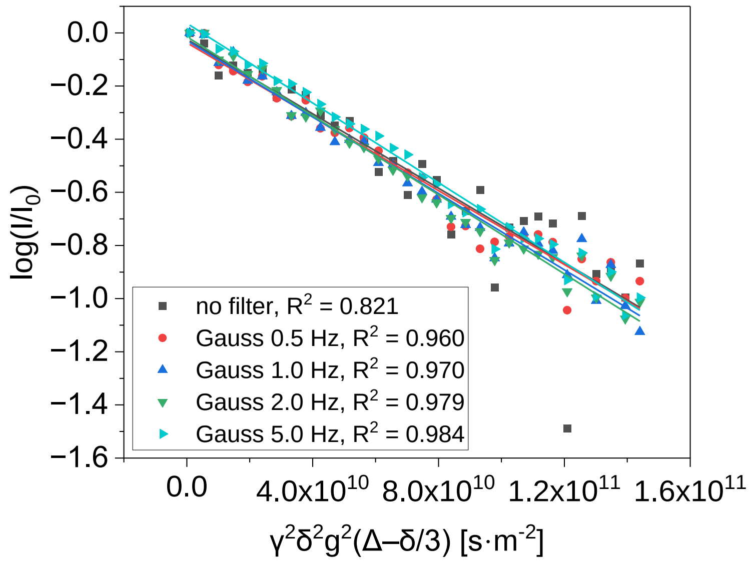

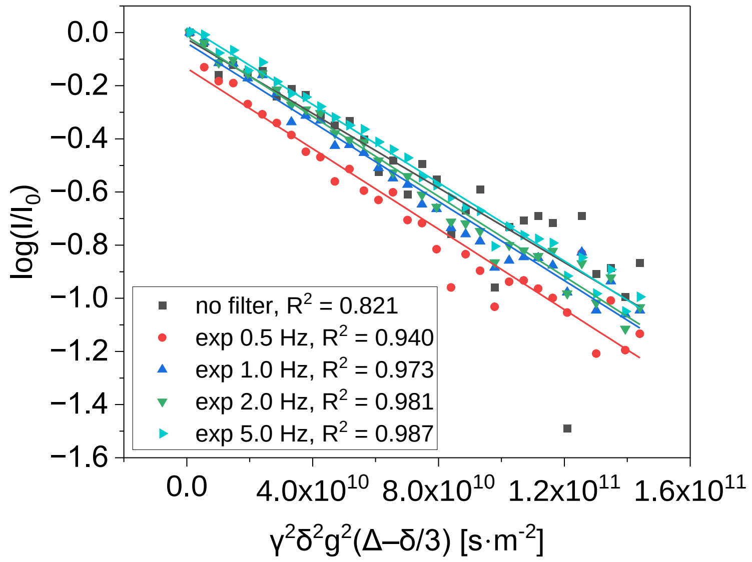

Figure S 7 Comparison of linear correlation (R^2) of Stejskal-Tanner plots of aromatic protons of PS550k. Processing was done with no, Gaussian (top), or exponential (bottom) filter functions at different standard deviations of the peak width: 0.5, 1.0, 2.0, 5.0 Hz. I/I_0 was *plotted on a logarithmic scale to measure the quality of the linear regression.*

3. Broadly distributed polystyrene sample

A polystyrene sample (PS133k) was synthesized in-house by radical bulk polymerization. Figure S8 compares the molar mass distributions obtained with SEC (reference), NMR-ILT, and NMR-log-normal with related molar masses and dispersities.

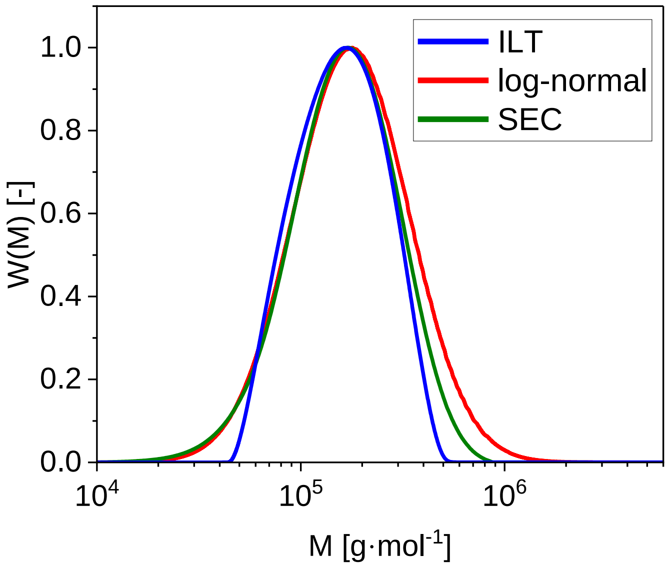

method	M_n [kg·mol^{-1}]	M_w [kg·mol^{-1}]	*Đ*
**SEC**	87	133	1.53
**NMR / ILT**	98	120	1.22
**NMR / Log-normal**	94	143	1.52

Figure S 8 Molar mass distribution of bulk polymerized PS133k, fitted with log-normal or inverse Laplace transformation (ILT). SEC served as a reference. Average molar masses and dispersities are shown in the table.

4. Dynamic light scattering (DLS)

The DLS measurement of sample PS123k was carried out with a Nicomp 380 instrument, equipped with a 12 mW laser (λ = 658 nm) and a detector angle of 90°. The viscosity of the deuterated chloroform was set to η = 0.54 mPa s and the temperature to 26 °C. The sample concentration was similar to that in NMR measurements with 2.5 g·L^{-1}. The channel width was set to 20 µs and the measurement was recorded for 15 minutes.

From the resulting autocorrelation function, the distribution of the hydrodynamic radius was modelled by an ILT using the analysis tool 'Nicomp Distribution' (version 1.99) implemented in the device. To obtain the distribution of the diffusion coefficients, the intensity-weighted data were first converted to number-weighted data and then converted with the Stokes-Einstein equation (equation 4, in main article). To represent the molar mass distribution, the diffusion coefficient distribution was transformed into the mass-weighted molar mass distribution using equation 6 and 9.

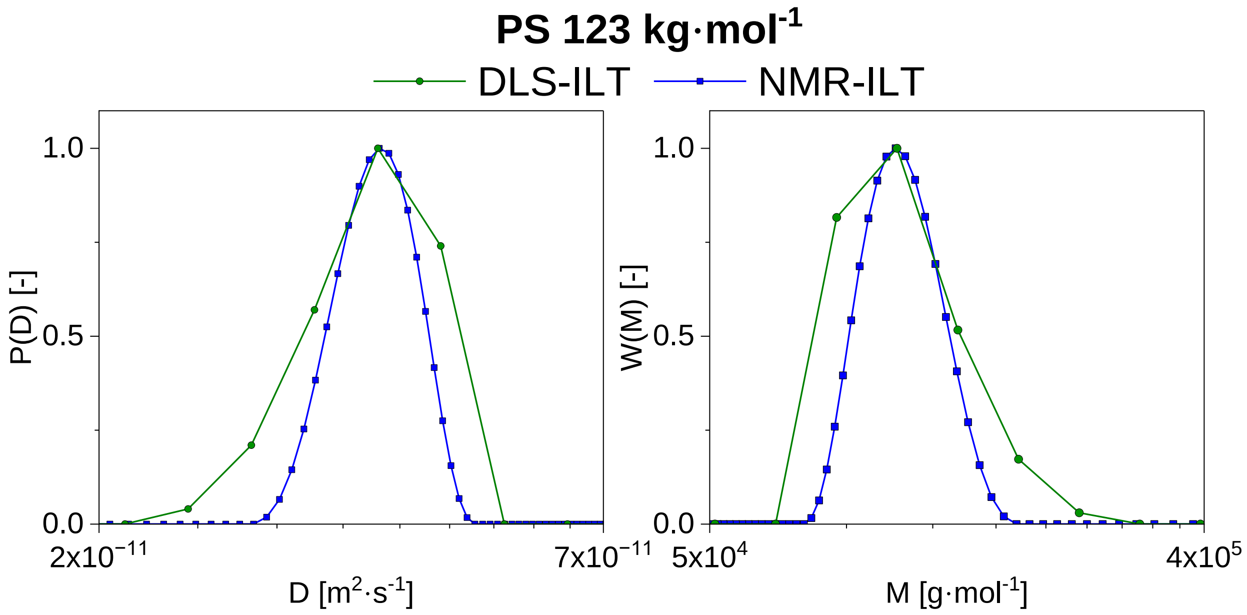

Figure S 9 Comparison of diffusion coefficient distributions (left) and molar mass distributions (right) of sample PS123k obtained from NMR and dynamic light scattering (DLS) with inverse Laplace transformation (ILT).

5. Characterisation of monomodal uniform PS samples

Table S 4 Molar masses obtained using either the monoexponential approach or an inverse Laplace transformation (ILT). Reference values from SEC are shown for comparison. Relative differences between experimental and reference values rel.diff. are listed in italics on the right of the corresponding experimental values.

sample	method	M_w [g·mol^{-1}]	*rel. diff. [%]*	M_n [g·mol^{-1}]	*rel. diff. [%]*	*Đ*
**PS10k**	SEC	9 460	-	9 220	-	1.03
	NMR / monoexp.	9 790	*3.49*	9 790	*6.18*	1.00
	NMR / ILT	9 720	*2.75*	9 624	*4.38*	1.01
**PS17k**	SEC	17 300	-	16 900	-	1.03
	NMR / monoexp.	17 000	*-1.73*	17 000	*0.59*	1.00
	NMR / ILT	19 000	*9.83*	18 700	*10.65*	1.02
**PS63k**	SEC	62 600	-	58 500	-	1.03
	NMR / monoexp.	61 200	*-2.24*	61 200	*4.62*	1.00
	NMR / ILT	63 700	*1.76*	61 400	*4.96*	1.04
**PS123k**	SEC	123 000	-	117 000	-	1.05
	NMR / monoexp.	117 000	*-4.88*	117 000	*0.00*	1.00
	NMR / ILT	116 000	*-5.69*	113 000	*-3.41*	1.03
**PS271k**	SEC	271 000	-	260 000	-	1.04
	NMR / monoexp.	244 000	*-9.96*	244 000	*-6.15*	1.00
	NMR / ILT	269 000	*-0.74*	256 000	*-1.54*	1.05
**PS516k**	SEC	516 000	-	451 000	-	1.14
	NMR / monoexp.	445 000	*-13.76*	445 000	*-1.33*	1.00
	NMR / ILT	510 000	*-1.16*	473 000	*4.88*	1.08
**PS931k**	SEC	931 000	-	790 000	-	1.18
	NMR / monoexp.	938 000	*0.64*	938 000	*18.61*	1.00
	NMR / ILT	1 240 000	*33.19*	985 000	*24.68*	1.26

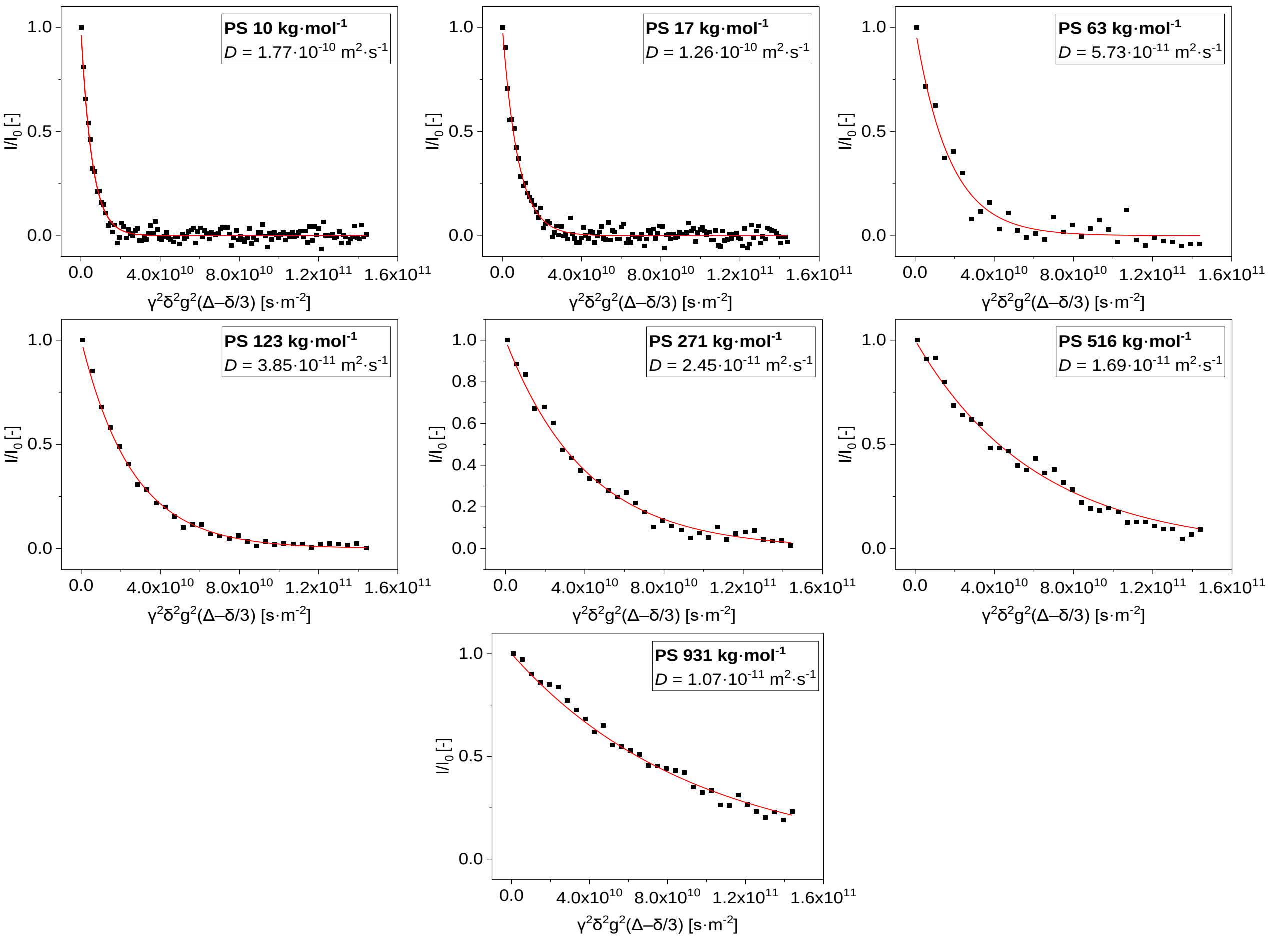

Figure S 10 Monoexponential fits of the intensities of the integrated aromatic proton signals of different PS samples.

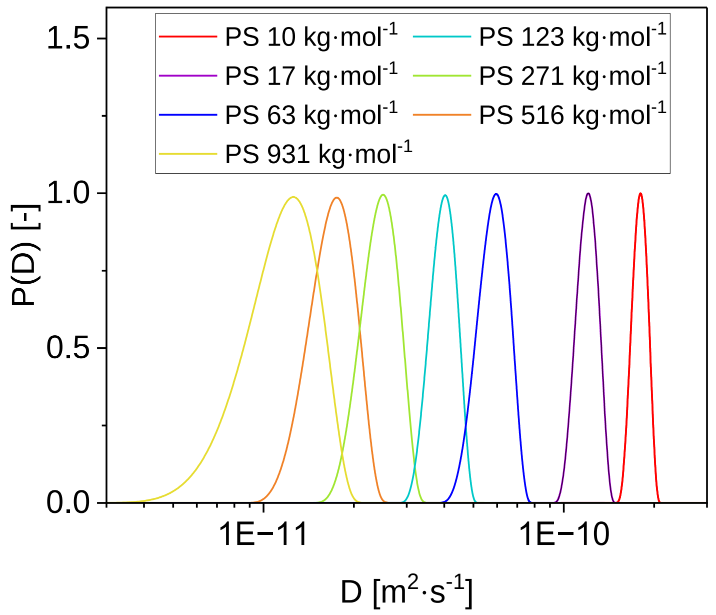

Figure S 11 Diffusion coefficient distributions obtained with inverse Laplace transformation of the intensities of the integrated aromatic proton signals of different PS samples.

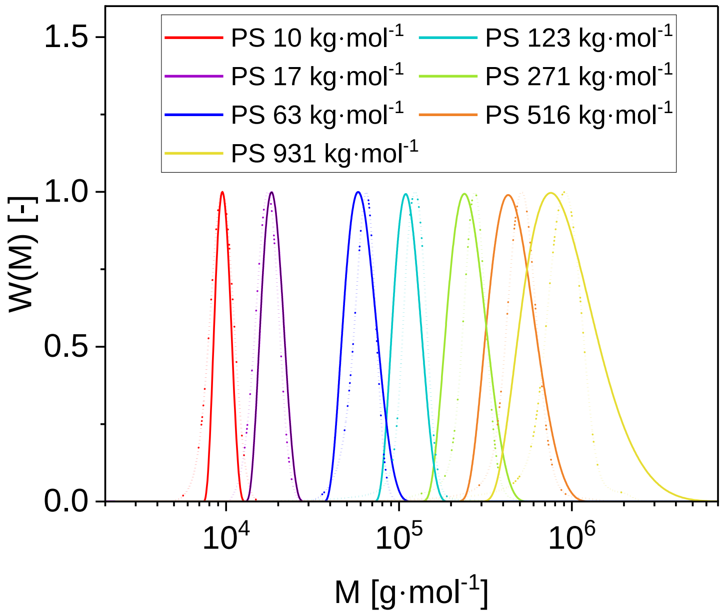

Figure S 12 Molar mass distributions obtained from diffusion coefficient distributions of different PS samples and determined with fitting parameters from PS calibration. Reference data obtained with SEC is shown in dotted lines, see Table S4 for numerical results.

6. Characterisation of bimodal PS samples

Table S 5 Molar masses (in kg·mol^{-1}) and relative weight fractions (in wt%) of bimodal PS samples measured with SEC or with NMR and calculated with either inverse Laplace transformation (ILT) or a biexponential fit. Reference values of weight fractions as prepared and molar masses of monomodal components are shown for comparison. Relative differences between bimodal sample experimental values and reference values rel.diff. are listed in italics on the right of the corresponding experimental values.

Monomodal components		Bimodal sample (blend of 2 monomodal components)											
		SEC				ILT				Biexponential fit			
M_w	X_{wt} a)	M_{wi}	rel.diff. [%]	X_{wt} b)	rel.diff. [%]	M_{wi}	rel.diff. [%]	X_{wt} b)	rel.diff. [%]	M_{wi}	rel.diff. [%]	X_{wt} b)	rel.diff. [%]
**10**	50	**9**	*-10*	53	*6.0*	**10**	*0.0*	49	*-2.0*	**9**	*-10*	55	*10*
**271**	50	**252**	*7.0*	47	*-6.0*	**295**	*8.8*	51	*2.0*	**290**	*7.0*	45	*-10*
**10**	30	**10**	*0.0*	34	*13*	**8**	*-20*	29	*-3.3*	**8**	*-20*	37	*23*
**271**	70	**256**	*5.5*	66	*5.7*	**276**	*1.8*	71	*1.4*	**252**	*-7.0*	63	*-10*
**10**	70	**10**	*0.0*	71	*1.4*	**10**	*0.0*	65	*-7.1*	**9**	*-10*	72	*-2.8*
**271**	30	**276**	*1.8*	29	*3.3*	**226**	*-17*	35	*17*	**208**	*-23*	28	*6.7*
**17**	50	**17**	*0.0*	52	*4.0*	**18**	*5.8*	54	*8.0*	**17**	*0.0*	58	*16*
**63**	50	**63**	*0.0*	48	*-4.0*	**72**	*14*	46	*-8.0*	**67**	*6.3*	42	*-16*
**10**	50	**9**	*-10*	45	*-10*	**6**	*-40*	92	*84*	**13**	*30*	96	*92*
**17**	50	**18**	*5.8*	55	*10*	**15**	*-12*	8	*-84*	**19**	*12*	4	*-92*

a) wt. fraction as prepared
b) wt. fraction as obtained from measurement/calculation

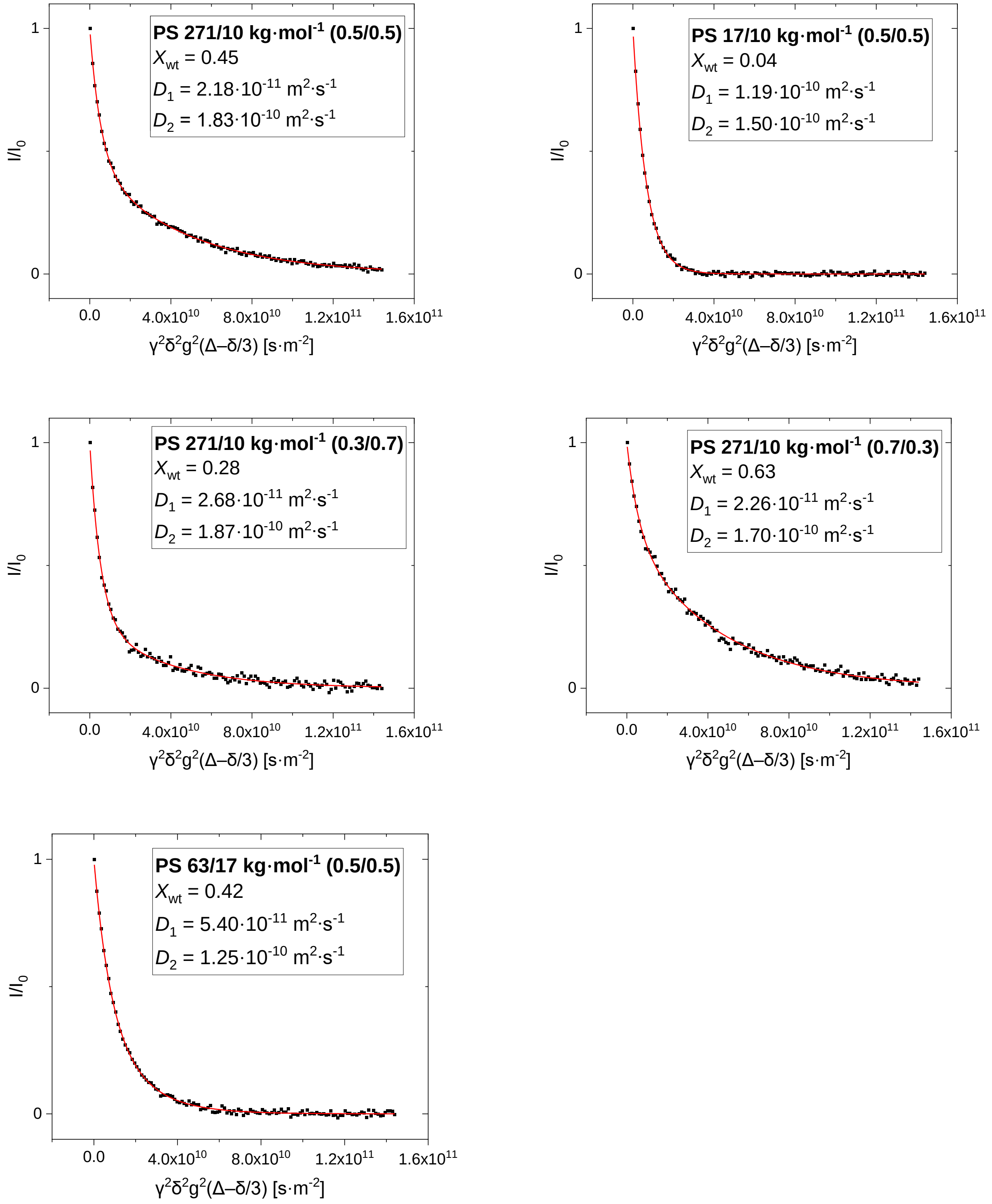

Figure S 13 Biexponential fits of the intensities of the integrated aromatic proton signals of PS blends with various molar masses and relative weights (X_{wt}).

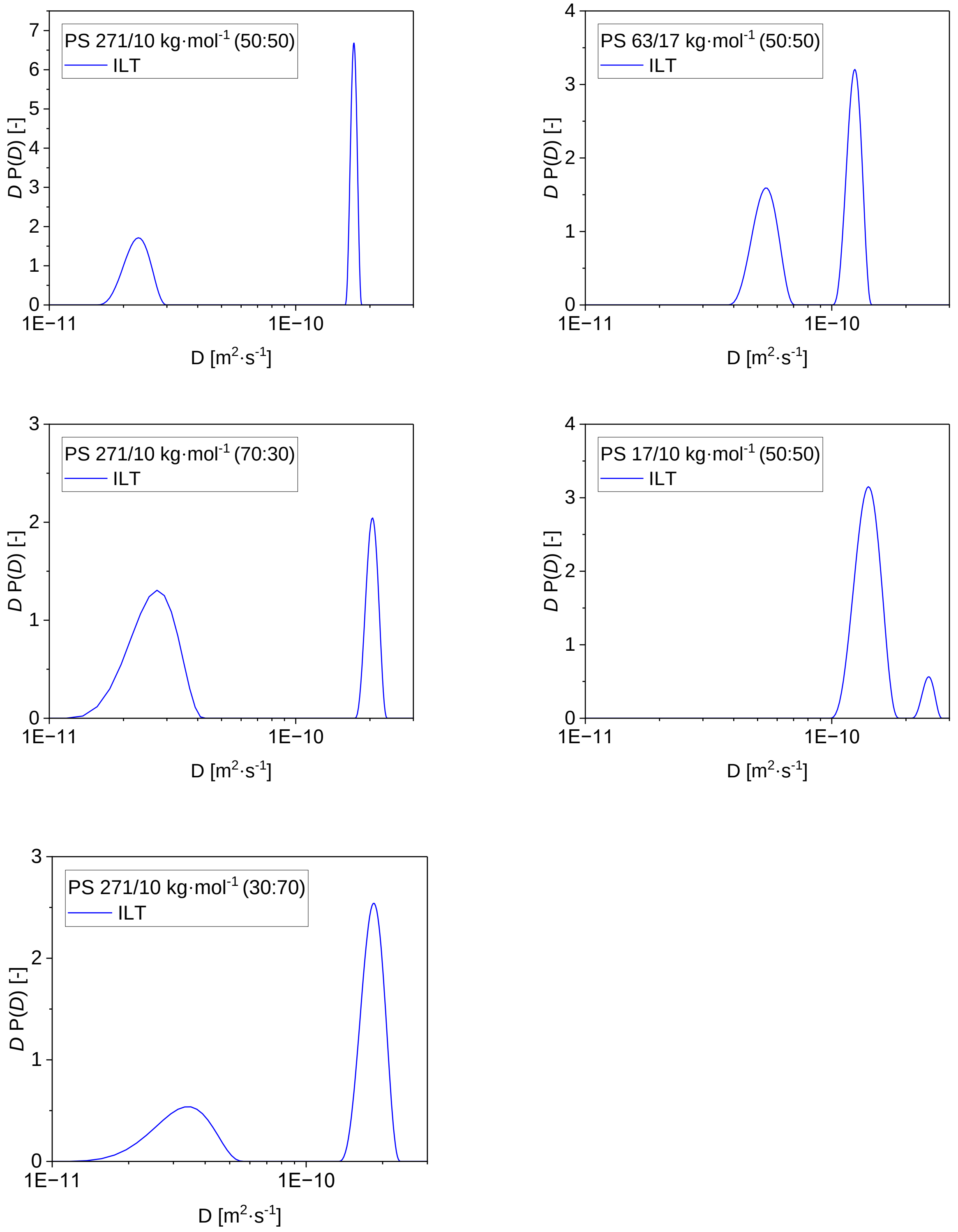

Figure S 14 Diffusion coefficient distribution obtained with inverse Laplace transformation of the intensities of the integrated aromatic proton signals of PS blends with various molar masses and relative weights.

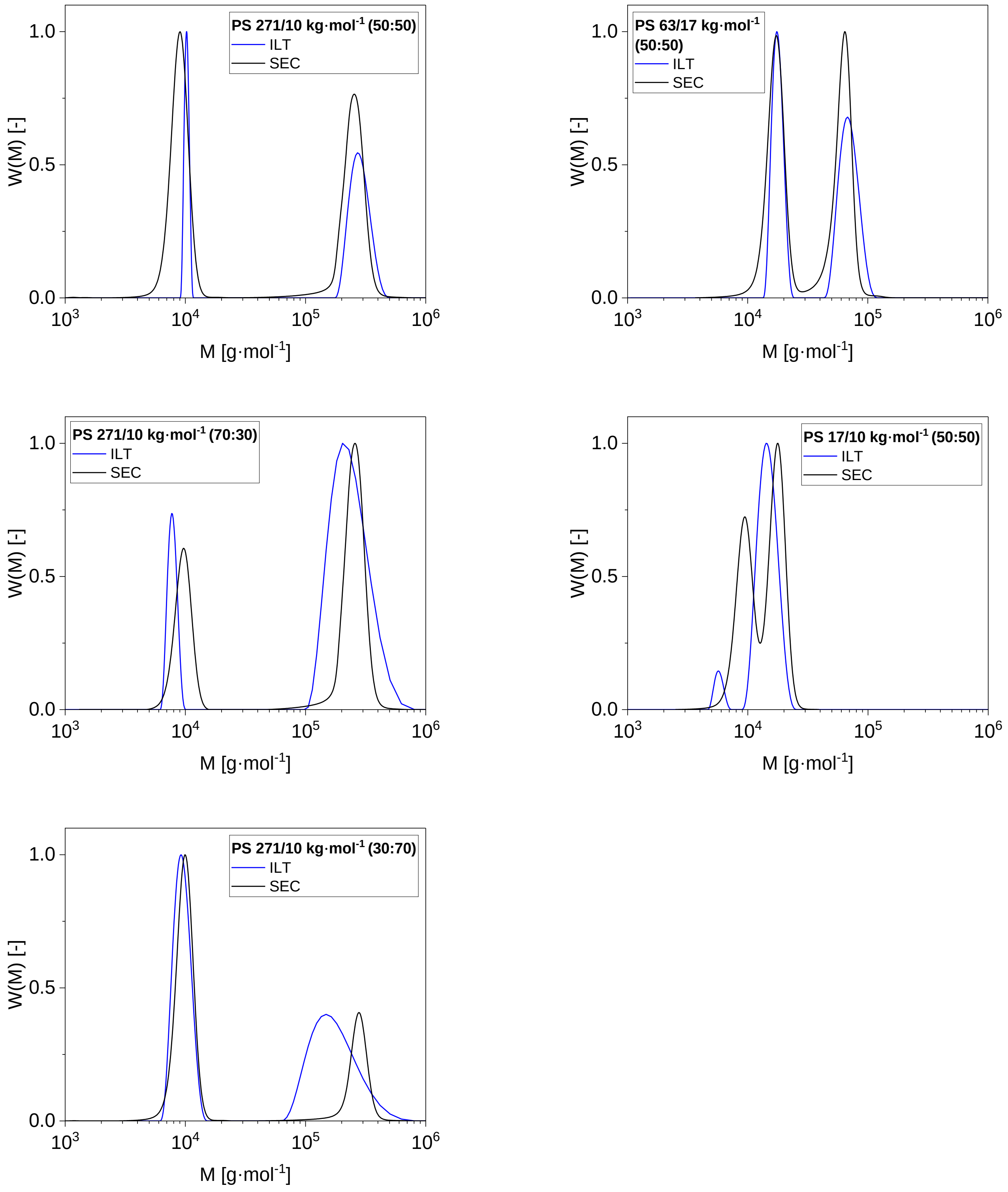

Figure S 15 Molar mass distributions obtained from diffusion coefficient distributions of different PS blends and determined with fitting parameters from PS calibration or obtained with SEC.

7. Characterisation of PS-b-PMMA block copolymers

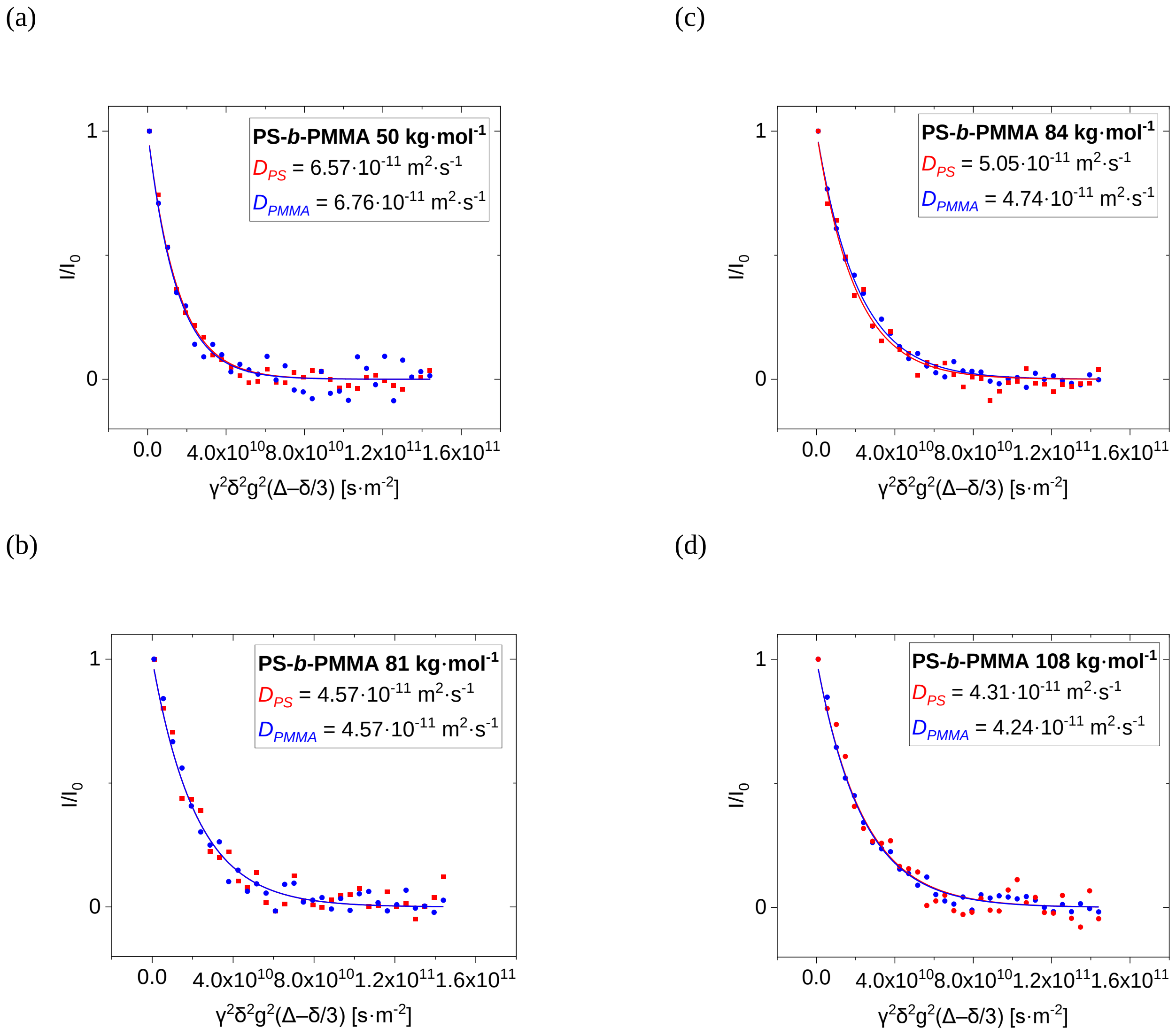

Figure S 16 Monoexponential fits of the intensities of the integrated methoxy group of PMMA (blue circles) and the aromatic protons of PS (red squares) for different PS-b-PMMA block copolymers: a) 50k, b) 81k c) 84k, d) 108k.

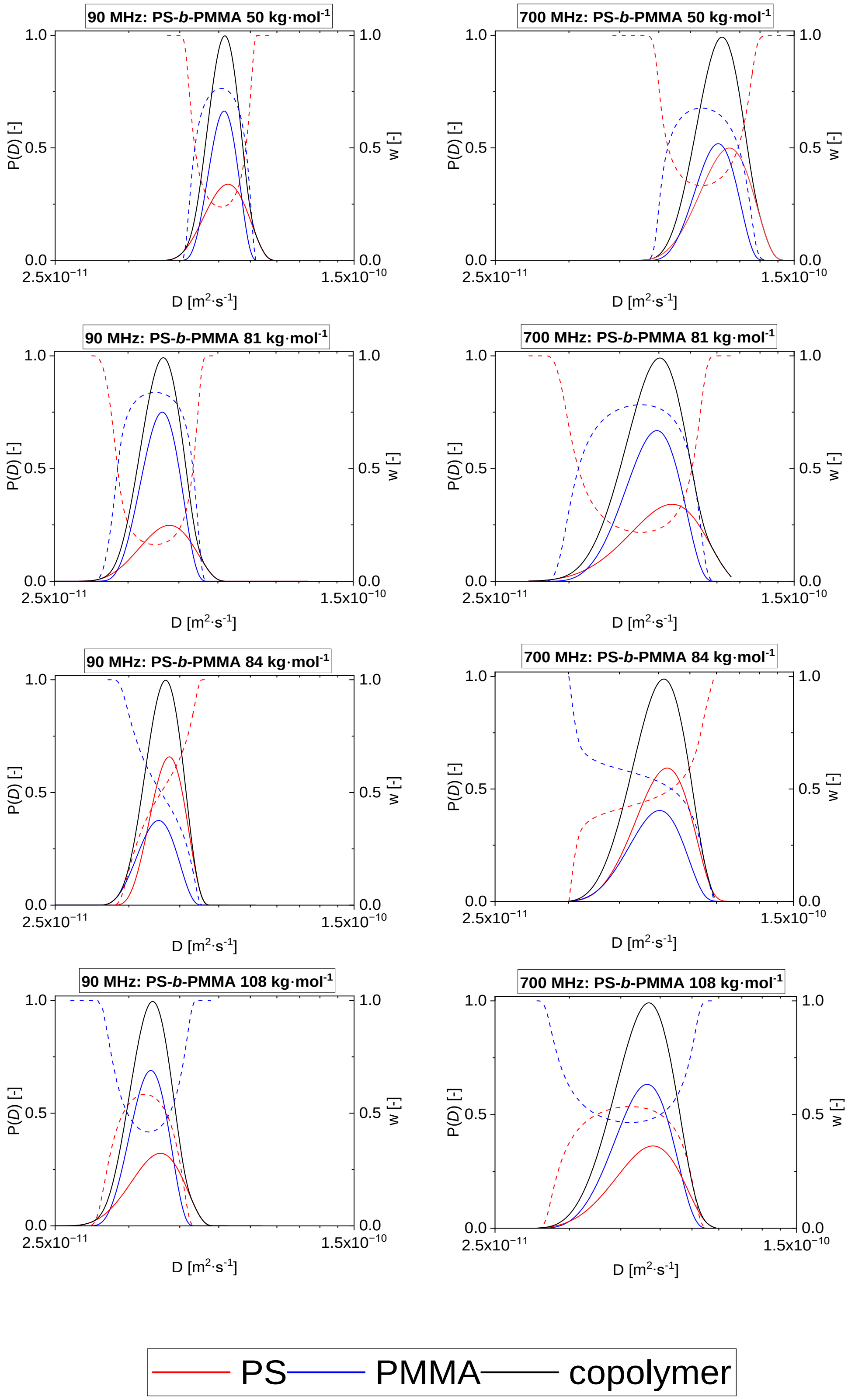

Figure S 17 Diffusion coefficient distributions (solid lines) and chemical compositions (dotted lines) obtained with inverse Laplace transformation of the intensities of the methoxy group of PMMA and the aromatic protons of PS of different PS-b-PMMA block copolymers. Graphs on the left were obtained with 90 MHz NMR, and graphs on the right were obtained with 700 MHz NMR. Data from 700 MHz NMR are from Bastian Grabe and Wolf Hiller, Macromolecules 2022, 55, 8014-8020, https://doi.org/10.1021/acs.macromol.2c01505.

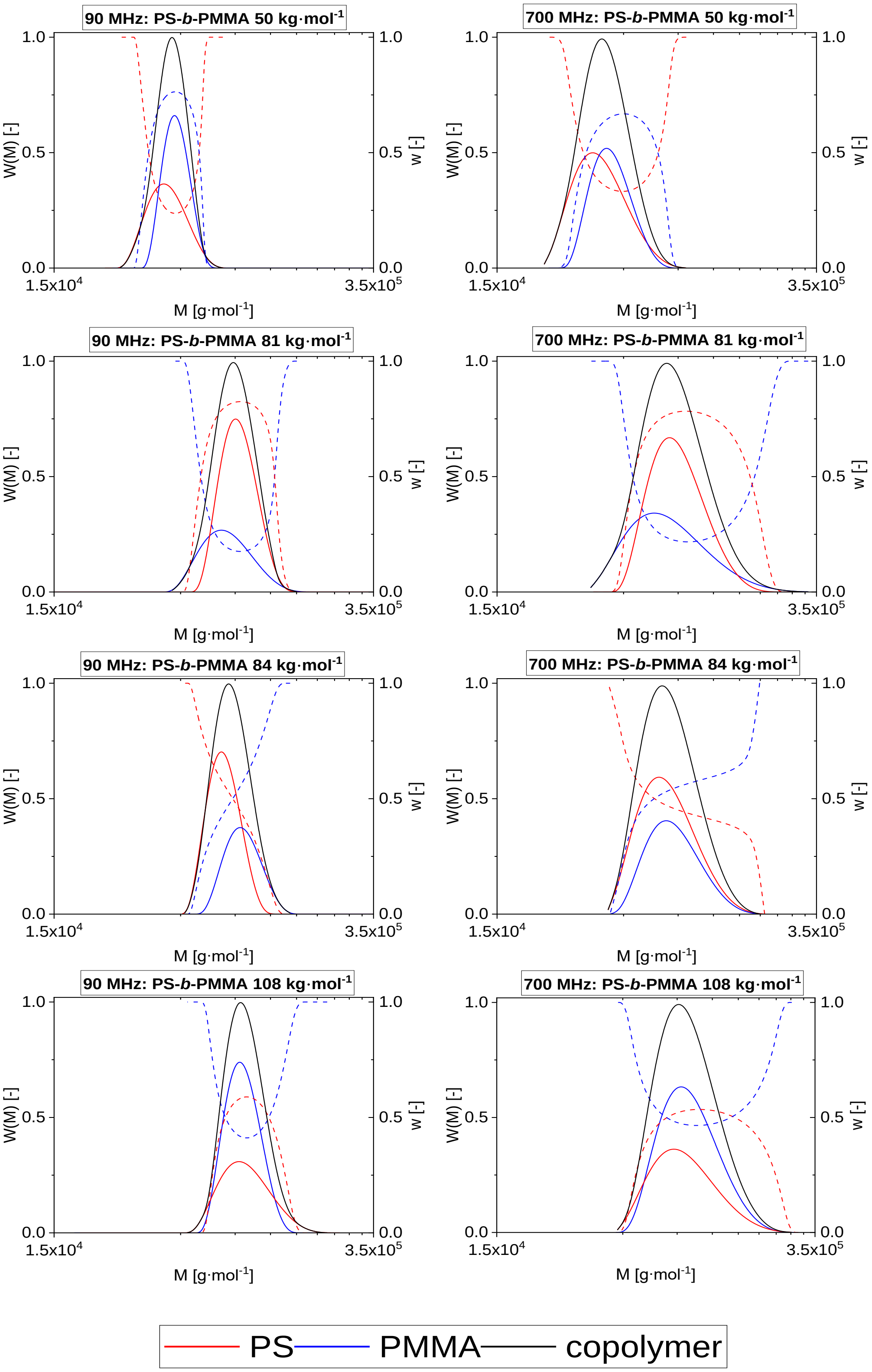

Figure S 18 Molar mass distributions (solid lines) and chemical compositions (dotted lines) obtained from diffusion coefficient distributions of different PS-b-PMMA block copolymers determined with fitting parameters from PS or PMMA calibrations. Graphs on the left were obtained with 90 MHz NMR, and graphs on the right were obtained with 700 MHz NMR. Data from 700 MHz NMR are from Bastian Grabe and Wolf Hiller, Macromolecules 2022, 55, 8014-8020, https://doi.org/10.1021/acs.macromol.2c01505.

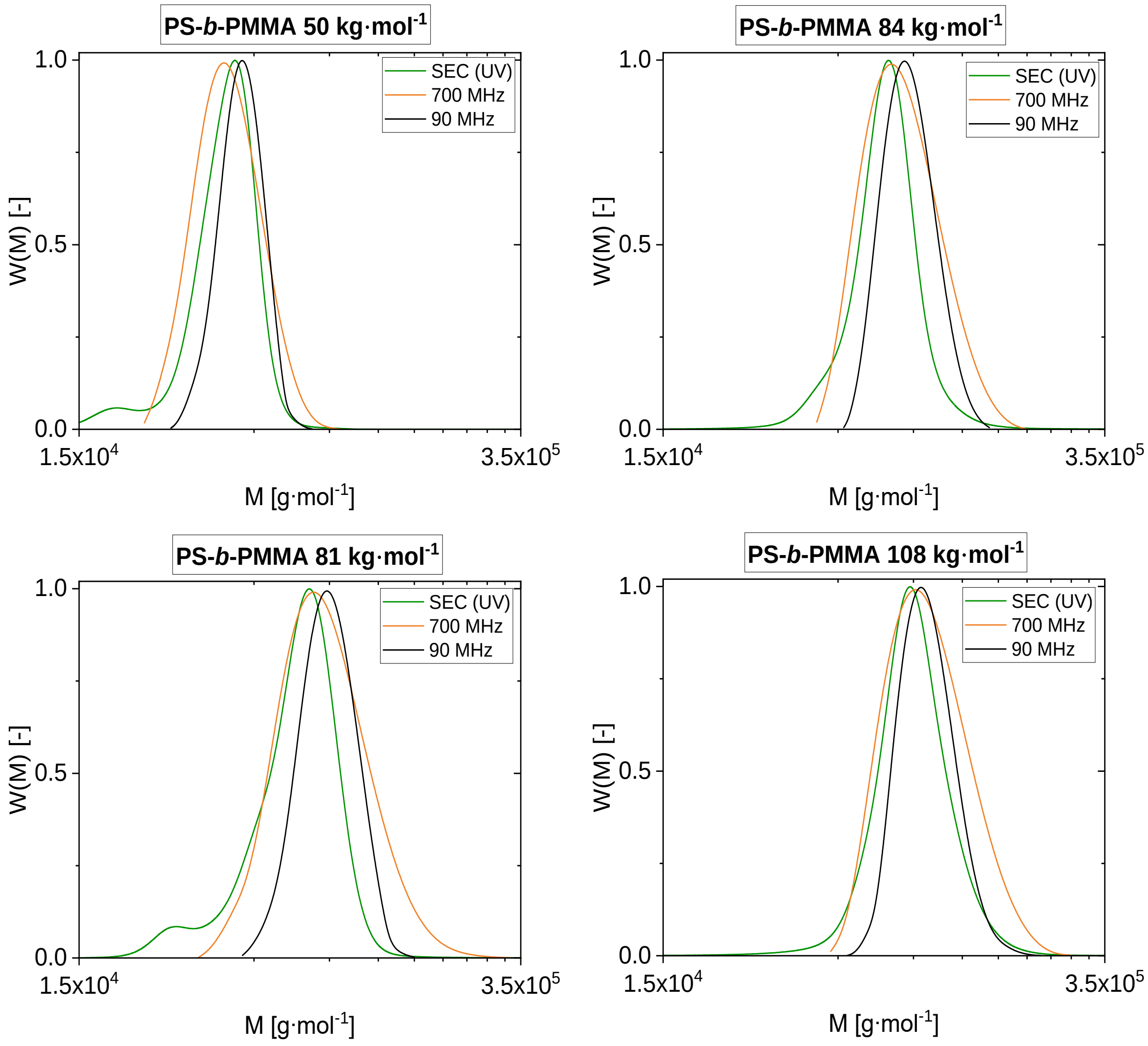

Figure S 19 Comparison of molar mass distributions of different PS-b-PMMA block copolymers obtained with SEC, 90 MHz DOSY-ILT and 700 MHz DOSY-ILT. Data from 700 MHz NMR was taken with the authors permission from Bastian Grabe and Wolf Hiller, Macromolecules 2022, 55, 8014-8020, https://doi.org/10.1021/acs.macromol.2c01505.

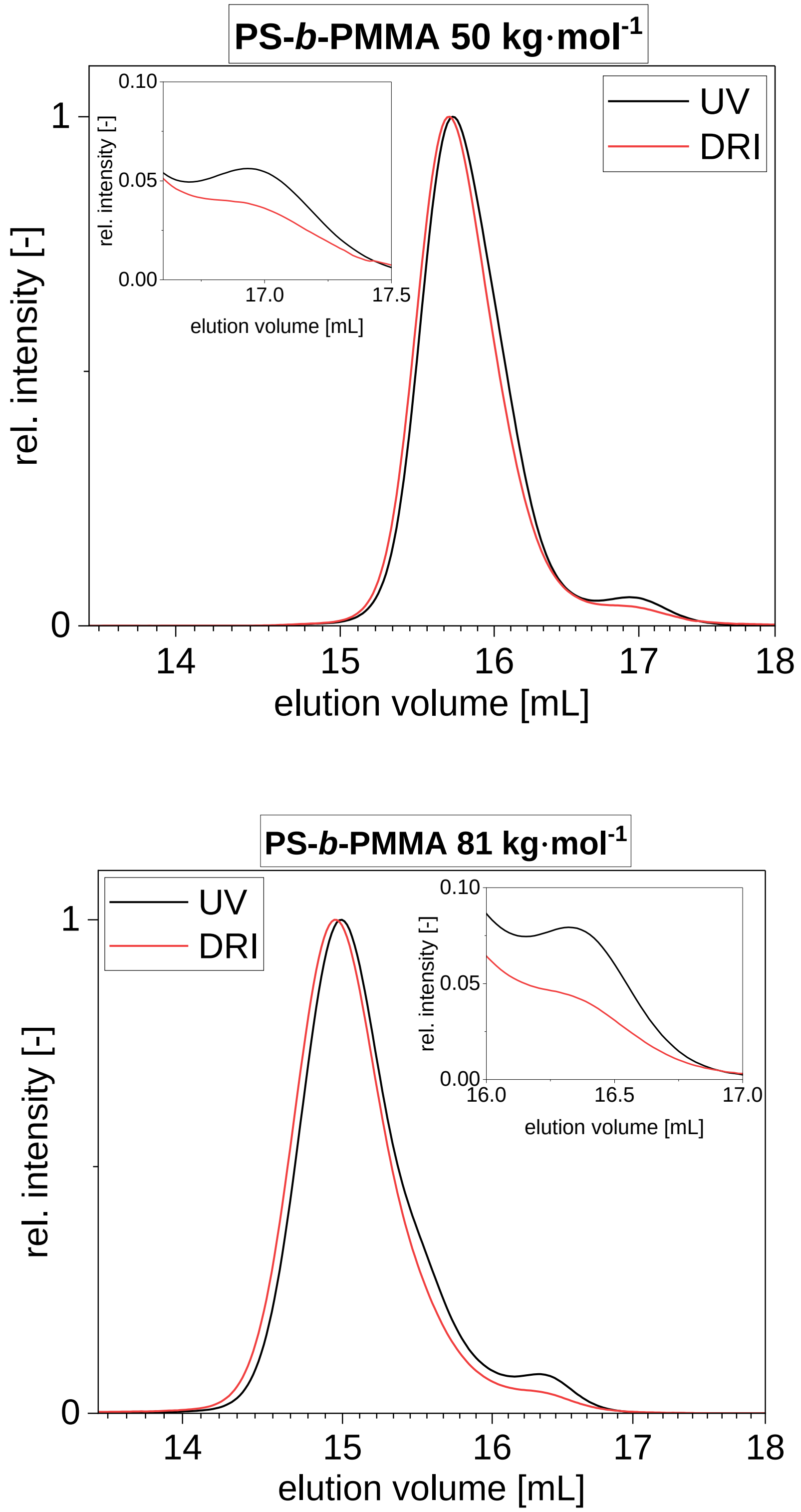

Figure S 20 SEC chromatograms of PS-b-PMMA 50 and 81 kg·mol^{-1}. The inset shows a zoom into the tailing region of the peaks.

8. Characterisation of PS in non-deuterated THF

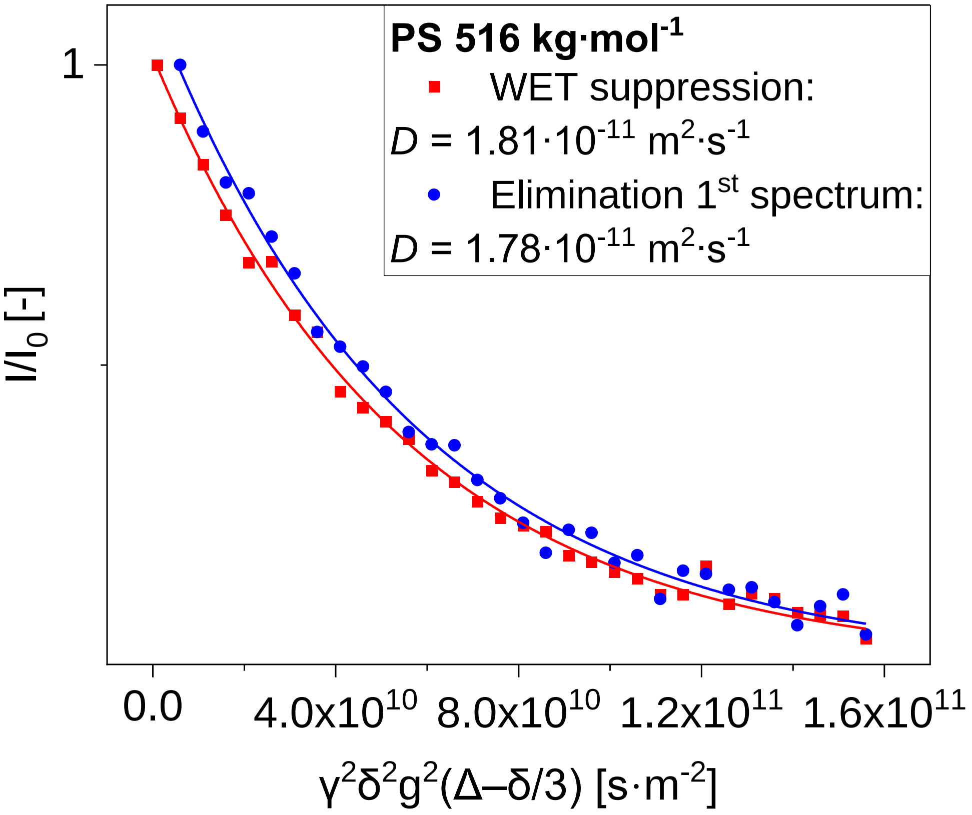

Figure S 21 Monoexponential fits of the intensities of the integrated aromatic proton signals of PS516k dissolved in protonated THF. Red squares show data obtained with WET solvent suppression, and blue circles show data without WET by eliminating the first data point (first gradient step).
